# Is blinding in studies of manual soft tissue mobilisation of the back possible? A feasibility randomised controlled trial with Swiss graduate students

**DOI:** 10.1186/s12998-023-00524-x

**Published:** 2024-01-29

**Authors:** Javier Muñoz Laguna, Emanuela Nyantakyi, Urmila Bhattacharyya, Kathrin Blum, Matteo Delucchi, Felix Karl-Ludwig Klingebiel, Marco Labarile, Andrea Roggo, Manuel Weber, Thomas Radtke, Milo A Puhan, Cesar A Hincapié

**Affiliations:** 1 EBPI-UZWH Musculoskeletal Epidemiology Research Group, University of Zurich, Zurich, Switzerland; 2 Epidemiology, Biostatistics and Prevention Institute (EBPI), University of Zurich, Zurich, Switzerland; 3 University Spine Centre Zurich (UWZH), Balgrist University Hospital, University of Zurich, Zurich, Switzerland; 4 Faculty of Medicine, Institute for Implementation Science in Health Care, University of Zurich, Zurich, Switzerland; 5 Institute of Evolutionary Medicine (IEM), University of Zurich, Zurich, Switzerland; 6 Centre of Computational Health, Institute of Computational Life Sciences, Zurich University of Applied Sciences (ZHAW), Zurich, Switzerland; 7 Department of Mathematics, University of Zurich, Zurich, Switzerland; 8 Department of Trauma, University Hospital Zurich, Zurich, Switzerland; 9 Harald Tscherne Laboratory for Orthopaedic and Trauma Research, University of Zurich, Zurich, Switzerland; 10 Division of Infectious Diseases and Hospital Epidemiology, University Hospital Zurich, Zurich, Switzerland; 11 Department of Dermatology, University Hospital Zurich, Zurich, Switzerland; 12 School of Health Professions, Academic-Practice-Partnership between Bern University of Applied Sciences and University Hospital of Bern, Bern University of Applied Sciences, Bern, Switzerland

**Keywords:** Methods, Blinding assessment, Manual therapy, Back pain, Double-blind method, Clinical trial

## Abstract

**Study design:**

Single-centre, two-parallel group, methodological randomised controlled trial to assess blinding feasibility.

**Background:**

Trials of manual therapy interventions of the back face methodological challenges regarding blinding feasibility and success. We assessed the feasibility of blinding an active manual soft tissue mobilisation and control intervention of the back. We also assessed whether blinding is feasible among outcome assessors and explored factors influencing perceptions about intervention assignment.

**Methods:**

On 7–8 November 2022, 24 participants were randomly allocated (1:1 ratio) to active or control manual interventions of the back. The active group (n = 11) received soft tissue mobilisation of the lumbar spine. The control group (n = 13) received light touch over the thoracic region with deep breathing exercises. The primary outcome was blinding of participants immediately after a one-time intervention session, as measured by the Bang blinding index (Bang BI). Bang BI ranges from –1 (complete opposite perceptions of intervention received) to 1 (complete correct perceptions), with 0 indicating ‘random guessing’—balanced ‘active’ and ‘control’ perceptions within an intervention arm. Secondary outcomes included blinding of outcome assessors and factors influencing perceptions about intervention assignment among both participants and outcome assessors, explored via thematic analysis.

**Results:**

24 participants were analysed following an intention-to-treat approach. 55% of participants in the active manual soft tissue mobilisation group correctly perceived their group assignment beyond chance immediately after intervention (Bang BI: 0.55 [95% confidence interval (CI), 0.25 to 0.84]), and 8% did so in the control group (0.08 [95% CI, −0.37 to 0.53]). Bang BIs in outcome assessors were 0.09 (−0.12 to 0.30) and −0.10 (−0.29 to 0.08) for active and control participants, respectively. Participants and outcome assessors reported varying factors related to their perceptions about intervention assignment.

**Conclusions:**

Blinding of participants allocated to an active soft tissue mobilisation of the back was not feasible in this methodological trial, whereas blinding of participants allocated to the control intervention and outcome assessors was adequate. Findings are limited due to imprecision and suboptimal generalisability to clinical settings. Careful thinking and consideration of blinding in manual therapy trials is warranted and needed.

**Trial registration:**

ClinicalTrials.gov: NCT05822947 (retrospectively registered)

**Supplementary Information:**

The online version contains supplementary material available at 10.1186/s12998-023-00524-x.

## Background

Manual therapy (MT) remains a guideline-compliant therapeutic option for back pain [1]. Yet, maintaining methodological quality in randomised controlled trials (RCTs) of MT interventions of the back poses challenges, particularly with respect to: (a) the design of a control (’sham‘) intervention and (b) the blinding status of participants and outcome assessors [2–4]. These challenges—compounded by poor reporting and contested blinding reporting standards [5]—can compromise internal validity via performance and detection biases [3]. Optimal implementation of high-quality MT trials requires assessing the feasibility of blinding participants and outcome assessors. Methodological trials focused on the assessment of blinding remain an opportunity for advancement in the field of MT RCTs [6–8].

The following methodological trial was conducted within the setting of a practice-based doctoral-level epidemiology course at the University of Zurich, Switzerland. Practice-based teaching methods are gaining traction in epidemiology curricula to foster skills among junior researchers in academic settings [9], including the development of scientific questions, and the planning, conduct, and analysis of RCTs. A multidisciplinary group of junior researchers was formed and challenged to design and execute a methodological RCT of MT interventions in a ‘learning-by-doing’ assignment—from research question formulation to final report and presentation of findings.

The primary objective of this methodological trial was to assess the feasibility of blinding an active manual soft tissue mobilisation and a control intervention of the back after a one-time intervention session. The secondary objective was to assess the feasibility of blinding the above interventions among outcome assessors and explore factors influencing perceptions about intervention assignment among participants and outcome assessors.

## Methods

### Study design and participants

The trial protocol along with the statistical analysis plan are available in Supplementary Material 1. This study was a two-parallel arm (allocation ratio 1:1), single-centre methodological RCT conducted among graduate students to assess the feasibility of blinding an active manual soft tissue mobilisation and a control intervention of the back. No changes were made to the methods after the launch of the trial. This manuscript was prepared in accordance with the 2010 Consolidated Standards of Reporting Trials (CONSORT) checklist extension for pilot and feasibility trials (Supplementary Material 2) [10].

This RCT included adults aged 18 years or older, enrolled in a practice-based doctoral-level epidemiology course at the University of Zurich (UZH), Switzerland. Research Electronic Data Capture (REDCap 12.5.14) was used to collect and store trial data.

Individuals were excluded if they reported pregnancy, had a serious pathology (i.e., cancer, severe scoliosis, inflammatory disease, infection, cauda equina syndrome or progressive motor deficit ≤ M3), a history of spine surgery, or an obvious contraindication to MT of the back (i.e., spinal fracture).

The independent research ethics committee of Canton Zurich (Kantonale Ethikkommission Zürich) deemed that approval was not required for this methodological trial of graduate students pursuant to Art. 2 (outside scope) of the Swiss Federal Act on Research involving Human Beings (Human Research Act, HRA). All participants provided electronic informed consent.

### Randomisation and blinding

Randomisation was computer-generated using permuted blocks of sizes two and four and stratified by ‘previous experience with MT’—operationalised as lifetime experience either providing MT as a healthcare professional or receiving MT in a healthcare setting. The allocation list was created by an independent biostatistician [11], and concealed within REDCap [12].

Study participants remained blinded to the primary trial objective, as the study information and consent form (Supplementary Material 3) masked the blinding assessment study aim. The study information form stated that the study aimed ‘to evaluate the effect of a MT intervention on back function by juxtaposing an active and control intervention’.

By nature of the intervention, intervention providers could not be blinded to intervention assignment, but were kept in a separate room. Intervention providers did not disclose the assigned intervention to participants or trial team members and performed randomisation immediately before intervention delivery. Outcome assessors, as well as data analysts remained blinded to the assigned intervention until analyses had been completed.

### Intervention procedures

Active manual soft tissue mobilisation and control interventions were designed to resemble each other in terms of participant-intervention provider interaction and duration (3 to 4 min). The active intervention involved a one-time session of mobilisation of the lumbar paraspinal musculature. With participants lying prone on a chiropractic table, the intervention provider applied hand-reinforced circumferential movements to six focal areas, using continuous ischemic compression strokes, and adjusting the pressure to participants’ tolerability (Supplementary Material 4, Figure S1). The active intervention was intentionally not designed to reflect a real-world clinical intervention by protocol. Yet, it contained an active element—a mechanical stimulus delivered to specific soft tissues with sufficient force and therapeutic intent [13]. The hypothesised mechanism of action at the soft tissue level was a decrease in muscle tone and stiffness leading to a potential increase in range of motion [13].

The control intervention included a one-time session of light touch to six distal, broad areas of the thoracic region, with a synchronised breathing exercise (Supplementary Material 4, Figure S1). The control intervention was not previously validated, although light touch in alternate areas is a common protocol in sham-controlled MT trials [3].

Both interventions were delivered by two members of the trial team after being trained in the intervention protocols by the corresponding author (JML, a Doctor of Chiropractic). Intervention providers were trained to standardise verbal and non-verbal cues (contextual effects) and followed a script for consistent interactions with participants.

### Range of motion assessment procedures

Three outcome assessors—Assessors 1, 2, and 3—were trained to measure range of motion (ROM) immediately before and after the one-time intervention session. ROM was measured standing by placing a mobile phone device at T12 using the iOS application Measure® (iOS version 16.0.2, iPhone® model X, Apple Inc., California), a suggested valid and reliable method [14–16]. Assessors 1 and 2 (‘measuring outcome assessors’) identified T12 through palpation and held the ROM measuring device in place as participants completed movements. Measuring outcome assessors were visually shielded from the measurement reading. Assessor 3 (‘documenting outcome assessor’) recorded measurement readings and was the only outcome assessor to actually see the ROM measurement values. A summary of the prespecified Standard Operating Procedures of the trial can be found in Supplementary Material 5.

### Outcomes

The prespecified primary outcome was blinding among participants immediately after a one-time intervention session, as measured by the Bang blinding index (see Table S1 in Supplementary Material 6 for relevant equations for Bang BI point estimate and variance) [17]. Data for the primary outcome were collected at the end of the post-intervention questionnaire, by asking participants: ‘To what extent do you know which intervention (active intervention or control intervention) you received?’. Possible responses were: ‘I strongly believe that I received the active intervention’, ‘I somewhat believe that I received the active intervention’, ‘I somewhat believe that I received the control intervention’, ‘I strongly believe that I received the control intervention’, and ‘I do not know’. The Bang BI ranges from –1 (complete opposite perception of intervention received) to 1 (complete correct perception of intervention received), with 0 indicating ‘random guessing’—balanced perceptions of ‘active’ and ‘control’ intervention received within an intervention arm. It can be interpreted as the proportion of participants who correctly perceived their intervention assignment within an intervention arm beyond chance. ‘Adequate blinding’ was operationalised as a Bang BI between –0.2 and 0.2 [18].

The arm-specific Bang BI point estimates and variances can be summed (BI_active_ + BI_control_) to measure the between-arm difference in proportions of the same intervention perception—obtaining a measure of study-level blinding. A summed Bang BI of 0 is desirable and generally implies an equal proportion of participants in both arms perceiving they received active intervention [19]. Values between –0.3 and 0.3 may suggest ‘adequate blinding’ (personal communication with Prof. Heejung Bang, 2 February 2023), although summed Bang BIs vary across interventions [19].

The first secondary outcome was blinding of participants, measured by the James BI [20]—an alternative measure of study-level blinding [7]—and calculated from the same data as the primary outcome. The James BI ranges from 0 (complete correct perceptions of intervention received) to 1 (complete ‘do not know’ perceptions), where 0.5 corresponds to 50% of perceptions being correct, and 50% incorrect. Lack of ‘adequate blinding’ is suggested when the upper bound of the two-sided confidence interval of the James BI is less than 0.5.

The next secondary outcome was blinding of outcome assessors immediately after the one-time intervention session following the Bang approach [17]. Data were collected by asking outcome assessors: ‘To what extent do you know which intervention (active intervention or control intervention) the participant received?’. Possible responses were: ‘I strongly believe that they received the active intervention’, ‘I somewhat believe that they received the active intervention’, ‘I somewhat believe that they received the control intervention’, ‘I strongly believe that they received the control intervention’, and ‘I do not know’.

Factors contributing to perceived intervention arm assignment among study participants and outcome assessors were explored with an open-ended question. Other outcomes were added to keep participants unaware of the blinding assessment trial objective. These included back function, operationalised as four items about ‘ache, pain, or discomfort’ (Cornell Musculoskeletal Discomfort Questionnaire), and self-reported back flexibility (International Fitness Scale) [21, 22]. Additionally, we incorporated a measure of maximum ROM in flexion and extension of the back.

### Statistical analysis

Given that the maximum sample size was fixed at the total number of students enrolled in the course (n = 26), a precision-based approach was used to consider sample size (i.e., width of the 95% confidence interval [CI]) for the arm-specific Bang BI estimates [23]. For a sample size of 26 participants, the 95% CI was the observed BI point estimate ± 0.315 points (0.63 points width of the 95% CI) for the group-specific Bang BI, according to Thompson’s method (Eq. 1) as described by Landsman and colleagues [24].

A concise statistical analysis plan was specified and developed a priori (Supplementary Material 1, section 10). To address the primary objective of the trial, blinding was analysed following an intention-to-treat approach. Descriptive statistics (medians and interquartile ranges for quantitative data and counts and percentages for categorical data) were calculated for all primary and secondary outcomes. No formal statistical tests of between-group differences were performed for back function or ROM outcomes, as this did not align with our primary objective. Factors contributing to perceptions about intervention arm assignment among study participants, as well as outcome assessors, were qualitatively analysed using an inductive approach. Following a pragmatic thematic analysis, three trial team members with experience in qualitative methods independently collated the responses and subsequently grouped them thematically by consensus [25]. All analyses were conducted using R [26], with use of the R package BI version 1.1.0 [27] to calculate BIs—both Bang and James.

## Results

**Fig. 1:**
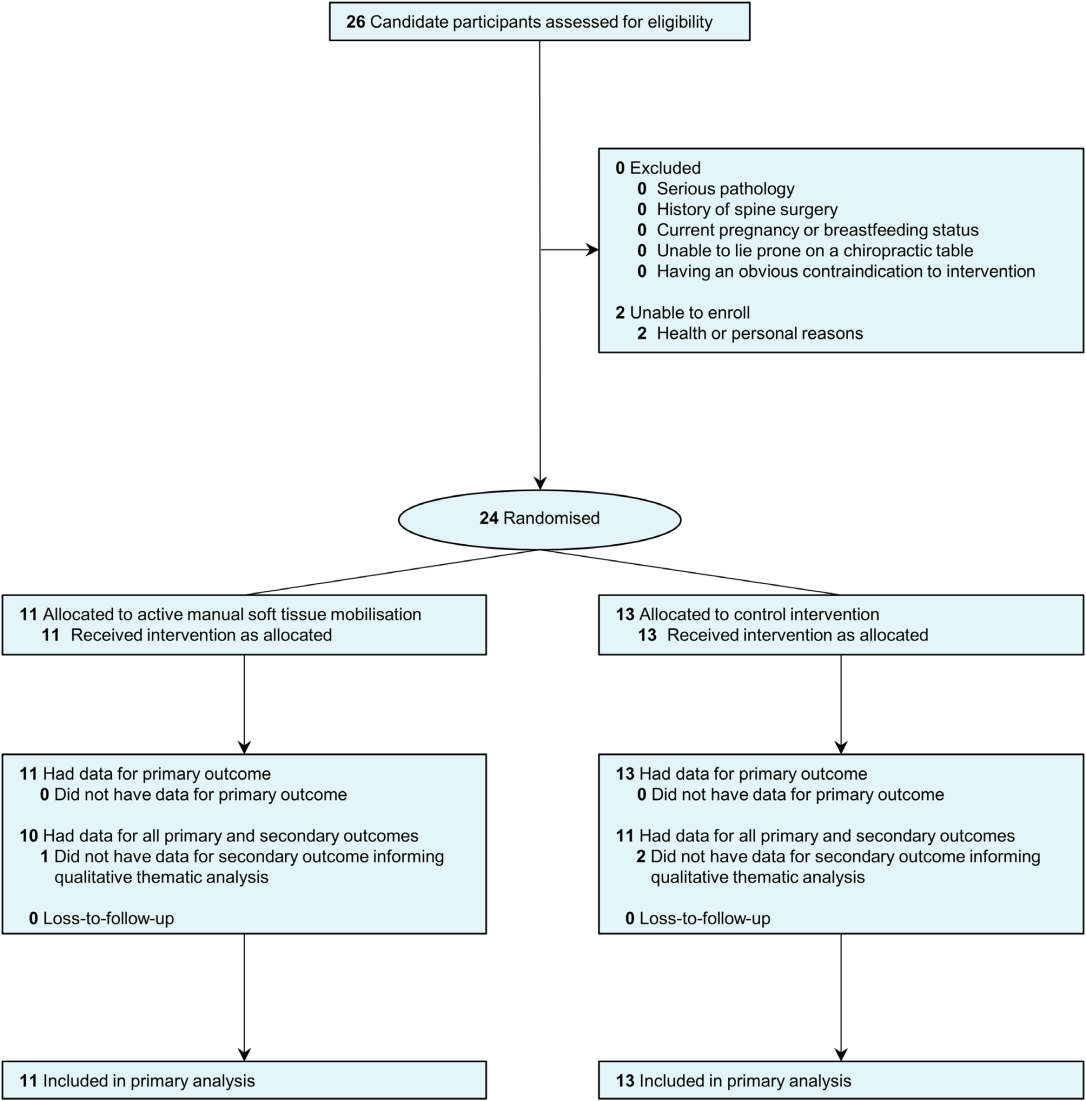
Participant recruitment, randomisation, and follow-up

26 students were approached and assessed for eligibility on November 7, 2022. 24 participants (active manual soft tissue mobilisation [n = 11]: median age [IQR], 28 [27 to 30] years, 64% women; control [n = 13]: median age [IQR], 28 [28 to 32] years, 77% female) were enrolled, randomised, and received their allocated intervention on November 8, 2022. There were no losses or exclusions after randomisation (Fig. 1). There were no missing data in the primary outcome. Both groups were comparable in most baseline characteristics (Table 1). Compared to the active manual soft tissue mobilisation, the control group had one more participant with MT experience and three more participants with self-reported ‘good or very good’ back flexibility (Table 1). Participants spent a median of 3.5 min (IQR, 3.2 to 4.1 min) receiving interventions and a median of 10.2 min (IQR, 9.9 to 10.3) for all trial procedures.

**Table 1:** Participant characteristics at baseline

Characteristic	Active manual soft tissue mobilisation (n = 11)	Control (n = 13)
Age — median (IQR)	28 (27 to 30)	28 (28 to 32)
Gender — N (%)		
Female	7 (64%)	10 (77%)
Male	4 (36%)	3 (23%)
Other or prefer not to say	0 (0%)	0 (0%)
Manual therapy experience — N (%)		
Providing only	0 (0%)	0 (0%)
Receiving only	2 (18%)	2 (15%)
Providing and receiving	1 (9%)	2 (15%)
No experience	8 (73%)	9 (69%)
Upper back ache, pain, or discomfort^a^ — N (%)		
None	9 (82%)	10 (77%)
Yes – slightly uncomfortable	2 (18%)	3 (23%)
Yes – moderately or very uncomfortable	0	0
Lower back ache, pain, or discomfort^a^ — N (%)		
None	10 (91%)	12 (92%)
Yes – slightly uncomfortable	1 (9%)	1 (8%)
Yes – moderately or very uncomfortable	0	0
Self-reported back flexibility^b^ — N (%)		
Good or very good	1 (9%)	4 (31%)
Average	9 (82%)	8 (62%)
Poor or very poor	1 (9%)	1 (8%)
ROM measurements (degrees) — median (IQR)		
ROM, flexion	127.0 (114.5 to 138.0)	132.0 (129.8 to 141.5)
ROM, extension	43.0 (27.5 to 53.0)	44.0 (33.8 to 52.0)

Abbreviations: *deg*, degrees; *IQR*, interquartile range; *ROM*, range of motion; *SD*, standard deviation

^a^ Adapted from the Cornell Musculoskeletal Discomfort Questionnaire [21]

^b^ Adapted from the International Fitness Scale [22]

### Blinding

#### Blinding of participants

Participant perceptions about intervention arm assignment, resulted in a Bang BI of 0.55 (95% CI, 0.25 to 0.84) in the active manual soft tissue mobilisation arm, and 0.08 (95% CI, -0.37 to 0.53) in the control arm (Table 2). These indices suggested that 55% of the active manual soft tissue mobilisation group correctly perceived their assigned intervention beyond chance, compared to 8% in the control group. Hence, the summed Bang BI was 0.63 (0.09 to 1.17). The James BI yielded an estimate of 0.53 (95% CI, 0.35 to 0.72). Full results of the blinding assessment in participants are provided in Supplementary Material 7, Table S2. Table S3 presents full results by levels of MT experience.

**Table 2:** Primary and secondary outcomes

Outcome	Active manual soft tissue mobilisation (n = 11)	Control (n = 13)	Effect (95% CI)
Primary outcome			
Bang BI in participants	0.55 (0.25 to 0.84)	0.08 (-0.37 to 0.53)	Summed^*^, 0.63 (0.09 to 1.17)
Secondary outcomes			
In participants			
James BI (n = 24)			0.53 (0.35 to 0.72)
Upper back ache, pain, or discomfort^a^—N (%)			
None	10 (91%)	10 (77%)	
Yes—slightly uncomfortable	1 (9%)	3 (23%)	
Yes—moderately or very uncomfortable	0	0	
Lower back ache, pain, or discomfort^a^—N (%)			
None	10 (91%)	12 (92%)	
Yes—slightly uncomfortable	1 (9%)	1 (8%)	
Yes—moderately or very uncomfortable	0	0	
Self-reported back flexibility^b^—N (%)			
Good or very good	5 (45%)	0	
Average	5 (45%)	8 (62%)	
Poor or very poor	1 (9%)	5 (38%)	
ROM measurements (degrees)—mean (SD)			
ROM, flexion	127.9 (18.7)	139.5 (11.5)	MD, -11.6 (-25.3 to 2.1)
ROM, extension	42.6 (13.6)	42.7 (15.2)	MD, -0.1 (-12.3 to 12.1)
ROM, total flex-ext	170.6 (27.7)	182.2 (21.6)	MD, -11.7 (-33.2 to 9.8)
Change in ROM, flexion	3.5 (7.9)	3.7 (4.3)	MD, -0.2 (-5.9 to 5.6)
Change in ROM, extension	2.0 (5.7)	1.5 (5.3)	MD, 0.5 (-4.2 to 5.2)
Change in ROM, total flex-ext	5.6 (9.3)	5.2 (7.7)	MD, 0.4 (-7.0 to 7.7)
In outcome assessors			
Bang BI—Assessor 1^†^	NaN	NaN	NaN
James BI—Assessor 1^†^			1 (1 to 1)
Bang BI—Assessor 2	0.27 (-0.09 to 0.63)	-0.23 (-0.54 to 0.08)	Summed^*^, 0.04 (-0.45 to 0.53)
James BI—Assessor 2			0.79 (0.65 to 0.93)
Bang BI—Assessor 3	0.00 (-0.50 to 0.50)	-0.08 (-0.53 to 0.37)	Summed^*^, -0.08 (-0.76 to 0.60)
James BI—Assessor 3			0.67 (0.48 to 0.85)
Bang BI—3 assessors	0.09 (-0.12 to 0.30)	-0.10 (-0.29 to 0.08)	Summed^*^, -0.01 (-0.29 to 0.27)
James BI—3 assessors (n = 24)			0.82 (0.73 to 0.90)

Abbreviations: *ext*, extension; *flex*, flexion; *MD*, mean difference; *NaN*, mathematically undefinable

^a^ Adapted from the Cornell Musculoskeletal Discomfort Questionnaire [21]

^b^ Adapted from the International Fitness Scale [22]

^*^ Sum of arm-specific Bang BIs—measures the difference in proportions of the same intervention perception [19]

^†^ All answers—‘I do not know’

### Blinding of outcome assessors

Bang BIs in outcome assessors were 0.09 (-0.12 to 0.30) and –0.10 (-0.29 to 0.08) for perceived assignment of active and control participants, respectively. The summed Bang BI for outcome assessors was –0.01 (-0.29 to 0.27). At the individual outcome assessor level, Bang BI estimates varied. Assessor 1 marked all responses as ‘I do not know’—this prevented any Bang BI calculation (mathematically undefinable). However, the interpretation of James BI for this assessor is compatible with optimal blinding (complete ambivalence). Assessor 2 had Bang BI estimates of 0.27 (95% CI, -0.09 to 0.64) and –0.23 (95% CI, -0.54 to 0.08) for the active and control arms, respectively. Assessor 3 had Bang BI estimates of 0.00 (95% CI, -0.50 to 0.50) and –0.08 (95% CI, -0.53 to 0.37). Table 2 presents BI estimates for outcome assessors. Full results of the blinding assessment in outcome assessors are provided in Supplementary Material 7, Table S4.

### Range of motion, back discomfort, and self-perceived flexibility

There were no between-group differences in any of the ROM outcomes (Table 2). Changes in back discomfort and self-perceived flexibility were comparable by levels of actual and perceived intervention assignment (Supplementary Material 7, Table S5).

### Factors contributing to perceived intervention assignment

Participants reported uncertainty regarding their perceived intervention assignment. Some mentioned that the applied manual pressure during the intervention as well as a perceived immediate intervention effect informed their justification of perceived intervention arm assignment. Other influencing factors included the use of breathing and contextual elements, such as the atmosphere during intervention delivery (Table 3).

Assessors 1 and 2 lacked certainty to justify their perceptions of intervention assignment among participants. In some instances, these assessors rationalised their choice based on participants’ verbal cues, speed or ease of movement, and perceived ROM. Assessor 3 justified most responses based on recorded ROM measurement readings.

**Table 3:** Factors contributing to perceptions about intervention arm assignment among participants and outcome assessors

Group (n, possible responses), Theme	n (%)	Findings summary	Selected verbatim (gender, age at interview, intervention assigned)
Study participants (n = 24)			
Uncertainty	5 (20.8%)	Participants expressed uncertainty regarding the intervention assignment, and the intervention comparison	‘I am not sure what the intervention was.’ (Female, 28 years old, control intervention)
Immediate effect	4 (16.7%)	Participants based their response on the perceived immediate effects after intervention	‘I felt tension release in the back musculature after the manipulation.’ (Female, 26 years old, active manual soft tissue mobilisation)
Manual pressure	4 (16.7%)	Participants based their response on manual pressure exerted by the intervention provider	‘The hand pressure felt too soft.’ (Female, 28 years old, control intervention)
Breathing	3 (12.5%)	Participants pointed to the associated breathing exercise of the intervention as the main element informing their response	‘Because a researcher forced breathing.’ (Female, 32 years old, control intervention)
Misconception	3 (12.5%)	Participants exhibited misconceptions surrounding the manual therapy intervention	‘I did not received [sic] an active massage, therefore I believe that I received the control intervention’ (Male, 29 years old, active manual soft tissue mobilisation)
Atmosphere	1 (4.2%)	Participant expressed expectations for certain contextual elements	‘There was no additional thing such as music.’ (Male, 36 years old, control intervention)
Misunderstanding	1 (4.2%)	Participants lacked understanding regarding the applied manual therapy intervention	‘I did not understand the intervention.’ (Male, 32 years old, active manual soft tissue mobilisation)
Non-response	3 (12.5%)	-	-
**Assessors 1 and 2—measuring outcome assessors (possible responses = 48)**			**Selected verbatim (identifier, intervention assigned to participant)**
Uncertainty	23 (47.9%)	Outcome assessors were uncertain or lacked confidence in their response	‘I don’t know.’ (Assessor 1, active manual soft tissue mobilisation)
Movement quality	5 (10.4%)	Outcome assessors pointed to certain movement quality elements, such as speed or stability	‘Shaking when leaning backwards (extension).’ (Assessor 2, active manual soft tissue mobilisation)
Range of motion	4 (8.3%)	Outcome assessors were under the impression that a change in range of motion had occurred	‘Impression: more degrees (flexion).’ (Assessor 2, active manual soft tissue mobilisation)
Verbal cues	2 (4.2%)	Outcome assessors took note of certain verbal cues by the participants when performing the assessments	‘I think participant thinks she got the treatment, said ‘it works’.’ (Assessor 1, control intervention)
Non-response	14 (29.2%)	-	-
**Assessor 3—documenting outcome assessor (possible responses = 24)**			**Selected verbatim (identifier, intervention assigned to participant)**
Movement quantity	16 (66.7%)	The outcome assessor compared pre- and post- measurements to justify response	‘Much improved flexibility in both directions.’ (Assessor 3, control intervention)
Uncertainty	8 (33.3%)	The outcome assessor expressed uncertainty about the pre- and post- measurements or had difficulty recalling the exact values	‘I don’t think there was a big change in measurement but not sure.’ (Assessor 3, control intervention)
Non-response	0 (0.0%)	-	-

### Adverse events

There was one mild adverse event reported in the control group immediately after the intervention—a transient exacerbation of an existing left subscapular complaint, which was deemed unrelated to the intervention.

## Discussion

### Major findings

In the present methodological trial, we assessed the feasibility of blinding an active manual soft tissue mobilisation and a control intervention of the back. We found that 55% of participants allocated to the active soft tissue intervention correctly identified their intervention assignment beyond chance level—suggesting that blinding in this group was not feasible. Our findings suggest that blinding of participants allocated to the control intervention and outcome assessors was adequate in our study.

The reported factors contributing to perceptions of the assigned intervention indicate that various aspects may influence blinding, including immediate intervention effects and manual pressure. These factors may vary based on trial roles.

### Comparison with existing evidence

Our Bang BI estimates for participants were similar to those of a recent meta-analysis of back pain RCTs [19]. Sham-controlled trials of MT for the back [28–31] have varied in their design of control interventions, including a range of light touch, drop table, and detuned instruments. Recent studies have also varied in their timing and blinding assessment methods, with a recent high-quality trial of MT [32] using the credibility/expectancy questionnaire [33].

### Ethical considerations

Methodological trials of blinding feasibility face ethical challenges with respect to the design of study information forms. In our study, the blinding assessment objective was not disclosed to participants until the end of the trial, which could have been considered a minor form of deception by some. However, by masking the study objective, the risk for positive or social desirability bias was mitigated [34]. Since the two interventions entailed minimal risks and full disclosure was provided at trial closure, we believe that according to Article 18 of the Swiss Human Research Act [35], the trial procedures were unlikely to be classified as involving incomplete study information. In addition, the research ethics committee of Canton Zurich deemed that approval was not required for this methodological trial pursuant to Art. 2 (outside scope) of the Swiss Federal Act on Research involving Human Beings (Human Research Act, HRA).

### Strengths and limitations

Our study has strengths. First, our RCT design maximised comparability between groups at baseline. Second, we maintained high quality during trial implementation and execution by concealing the allocation sequence, having no deviations from our prespecified protocol, benefitting from no missing data in our primary outcome, choosing a validated and following a standardised method for outcome measurement, analysing our trial results in accordance with our prespecified statistical analysis plan, and reporting on all of our prespecified outcomes [36]. Third, our assessment of blinding extended beyond participants and included outcome assessors, who are often neglected in blinding assessments. Fourth, to our knowledge, this is the first study to include a qualitative exploration of factors influencing perceptions about intervention assignment—an approach that can help to inform the design and procedures of future MT trials.

Our study has limitations. First, we were restricted to a small sample of doctoral students, which limited the precision and generalisability of our BI estimates. Second, our methodological trial was conducted in a non-clinical setting and interventions were delivered by persons with minimal MT training. Future blinding feasibility trials of MT should expand this preliminary work in clinical populations and settings [37]. Third, by conducting a single intervention session and assessing blinding at one time point, we were unable to evaluate possible temporal effects on blinding. Despite their potential added value, longitudinal assessments of blinding may be prone to evolving ‘hunches’ (i.e., perceptions about intervention assignment influenced by multiple treatment sessions and effects) [6, 38]. Fourth, our control intervention was not validated and the possibility that it contained active therapeutic components cannot be ruled out. The appropriateness of our control intervention may have been influenced by our beliefs about what constituted the active element of the manual soft tissue mobilisation intervention [39]. Fifth, our blinding assessment was restricted to the evaluation of perceptions about intervention assignment and did not capture other recommended constructs (e.g., credibility or expectancy [33]) relevant in sham-controlled trials [40].

## Conclusion

Blinding of participants allocated to an active soft tissue mobilisation of the back was not feasible in this methodological trial. However, blinding of participants allocated to the control intervention and outcome assessors was adequate. Factors contributing to perceptions about intervention assignment provide valuable information for future trial methods and blinding approaches. Careful consideration and assessment of blinding in MT trials is warranted and needed.

### Electronic supplementary material

Below is the link to the electronic supplementary material.

**Supplementary Material 1:** Trial protocol

**Supplementary Material 2:** 2010 CONSORT checklist

**Supplementary Material 3:** Study information and consent form

**Supplementary Material 4:** Figure S1

**Supplementary Material 5:** Standard Operating Procedures

**Supplementary Material 6:** Table S1

**Supplementary Material 7:** Tables S2, S3, S4, and S5

## Data Availability

The collected data files and other materials are available on reasonable request from the corresponding author.

## List of abbreviations

BI: Blinding index
MT: Manual therapy
ROM: Range of motion
UZH: University of Zurich
