# Supplementary material for: Is blinding in studies of manual soft tissue mobilisation of the back possible? A feasibility randomised controlled trial with Swiss graduate students
Source: Chiropr Man Therap. 2024 Jan 29;32:3. doi: 10.1186/s12998-023-00524-x (PMC10826218; doi:10.1186/s12998-023-00524-x)
Supplement: Supplementary file 1 — Supplementary Material 1: Trial protocol [file 12998_2023_524_MOESM1_ESM.pdf]

Clinical Study Protocol

Assessing manual interventions of the back in Swiss graduate students: a randomized controlled trial (SENSATE)

Study Type:	Other Clinical Trial
Study Categorization:	Other Clinical Trial Category *A (low risk)*
Study Registration:	Intended registration in clinicaltrials.gov
Study Identifier:	SENSATE
Sponsor-Investigator and Principal Investigator:	Javier Muñoz Laguna, DC MSc<br>EPI 301 Introduction to Epidemiology Course<br>Epidemiology, Biostatistics and Prevention Institute<br>University of Zurich
Study Intervention:	Active spinal manual therapy versus sham spinal manual therapy
Protocol Version and Date:	Version 1.0 (dated 04.11.2022)

PREAMBLE

This study will be conducted in the context of an academic environment as part of the course requirements for the EPI301 Introduction to Epidemiology graduate level course, in the Fall 2022 semester, at the University of Zurich, Zurich, Switzerland. Some aspects of this Clinical Study Protocol are illustrative in nature.

TABLE OF CONTENTS

LIST OF ABBREVIATIONS 3
1 ETHICAL AND REGULATOR ASPECTS 4
1.1 Study Registration 4
1.2 Competent Ethics Committee (CEC) 4
1.3 Ethical Conduct of the Study 4
1.4 Declaration of Interest 4
1.5 Participant Information and Informed Consent 4
1.6 Participant Privacy and Confidentiality 4
2 INTRODUCTION 5
2.1 Background and Rationale 5
2.2 Justification of Study Intervention 6
2.3 Explanation for Choice of Comparator Intervention
2.4 Risk / Benefits 6
2.5 Rationale for Chosen Study Population 6
3 STUDY OBJECTIVES 7
3.1 Primary Objective 7
3.2 Secondary Objectives 7
4 STUDY OUTCOMES 7
4.1 Primary Outcome 7
4.2 Secondary Outcomes 7
4.3 Safety Outcomes 7
5 STUDY DESIGN AND COURSE OF STUDY 7
5.1 General Study Design and Justification of the Design 7
5.2 Study Duration and Study Schedule 8
5.3 Methods of Minimizing Bias 8
6 STUDY POPULATION 8
6.1 Eligibility Criteria 9
6.2 Recruitment and Screening 9
6.3 Criteria for Withdrawal/ Discontinuation of Participants 9
7 STUDY INTERVENTION 9
7.1 General Information 9
7.2 Compliance with Intervention 10
7.3 Data Collection and Follow-up for Withdrawn Participants 10
8 STUDY PROCEDURES 11
8.1 Study Flow Chart/Table of Study Procedures and Assessments 11
8.2 Assessments of Outcomes 13
8.3 Assessment of Safety Outcomes 13
8.4 Procedures at Each Visit 14
9 SAFETY 14
9.1 Definitions 14
9.2 Recording and Assessment of Serious Adverse Events 15
9.3 Reporting of Serious Adverse Events 15
10 STATISTICAL METHODS 15
10.1 Sample Size Consideration 15
10.2 Planned Analyses 16
10.3 Handling of Missing Data and Drop-Outs 16
11 REFERENCES 17

LIST OF ABBREVIATIONS

AE	Adverse Event
BI	Blinding Index
CEC	Cantonal Ethics Committee
CRF	Case Report Form
eCRF	Electronic Case Report Form
EBPI	Epidemiology, Biostatistics and Prevention Institute
GCP	Good Clinical Practice
ICH	International Council on Harmonization
ISF	Investigator Site File
KEK	Kantonale Ethikkommission
LBP	Low Back Pain
MSK	Musculoskeletal
MT	Manual Therapy
PI	Principal Investigator
RCT	Randomized Controlled Trial
REDCap	Research Electronic Data Capture
ROM	Range of Motion
SAE	Serious Adverse Event
SNCTP	Swiss National Clinical Trial Portal
SOP	Standard Operating Procedure
TMF	Trial Master File

1 ETHICAL AND REGULATOR ASPECTS

Before the start of the study, the protocol, proposed participant information and consent forms, as well as other study-specific documents would be submitted to a properly constituted Competent Ethics Committee (CEC) in agreement with local legal requirements, for formal approval or formal justified exemption, if applicable.

Since this blinding feasibility trial involved an intervention that is neither a cure, a transplant product, nor a transplant, it would be expected to fall into the low-risk category, Category A. The clinical study would only begin once approval from the CEC has been received.

1.1 Study Registration

This study will be conducted in the context of an academic environment and will therefore not be formally uploaded to a registry. However, if the study were to be registered, the following registries would be useful: Swiss National Clinical Trials Portal (SNCTP) and clinicaltrials.gov.

1.2 Competent Ethics Committee (CEC)

For the purposes of this study, the Kantonale Ethikkommission (KEK) of the Canton of Zurich would serve as CEC.

1.3 Ethical Conduct of the Study

The study will be carried out in accordance with principles outlined in the current version of the Declaration of Helsinki, Good Clinical Practice (GCP) issued by International Council on Harmonization (ICH), and Swiss competent authority's requirements.

1.4 Declaration of Interest

The authors have no competing interests to declare.

1.5 Participant Information and Informed Consent

All study participants will receive a detailed participant information and consent form. The nature of the study, its purpose, the two trialed interventions, expected duration, potential risks, and any potential discomfort will be explained therein. All information will be provided without compromising the primary objective of evaluating blinding success. Although conceptualized in the context of a graduate-level class at the University of Zurich (Introduction to Epidemiology, EPI301, Fall 2022), participation in the proposed study is voluntary and participants can withdraw at any time. Completed formal informed consent forms are required in order to proceed with study participation.

1.6 Participant Privacy and Confidentiality

The investigators are responsible for treating all information related to the study and the compiled data with strict confidentiality. No information will be distributed to entities not directly involved with the study. Data generation, transmission, archiving, and analysis of personal data within this study strictly follow the current Swiss legal requirements for data protection. Non-participation or withdrawal will not entail any disadvantages.

All medical information obtained for this study is considered confidential and disclosure to third parties is prohibited. Participant confidentiality will be further ensured by using participant identification codes that correspond with intervention data in electronic files, following a well-defined standard operating procedure (SOP).

Data generated as a result of this study will be made available for inspection on request by the assigned trial monitors and by the KEK.

2 INTRODUCTION

Methodological research efforts have focused on improving mechanisms and advancing techniques for blinding and allocation concealment processes in pharmacological trials, however maintaining such standards in high-quality randomized controlled clinical trials (RCTs) for non-pharmacological interventions has proven difficult.[1,2] The concept of blinding describes the ignorance of study participants and/or study investigators to allocated intervention arms *during* the entire study period, while allocation concealment specifically describes the allocation of participants being concealed *before* inception of the study. Bias can happen at different stages of study execution; however effective blinding can minimize their occurrence. Exemplary types include misclassification bias (i.e., bias resulting from wrongful allocation of participants to intervention arms), performance bias (i.e., bias resulting from knowledge of applied interventions in either study investigators or participants), or detection bias (i.e., bias effects during outcome measurements).[3,4]

Clinical trials of manual therapy (MT) interventions, although considered the reference standard for testing efficacy, face methodological challenges in the design of successful shams and blinding of assigned interventions.[5] The use of a valid sham as a comparator in explanatory trials aims to discriminate specific from non-specific effects (i.e., contextual, not depending on the intervention itself) and mitigate performance, ascertainment, information, and other biases.[6] Despite consensus on the ideal design of credible shams in physical interventions for pain and the proposal of some creative sham interventions (i.e., detuned laser therapy or ultrasound, deactivated instruments, low-velocity maneuvers in alternative body areas), few state-of-the-art clinical trials have assessed successful intervention blinding between active and control participants.[7] This trend becomes more surprising when considering emerging evidence supporting the idea that clinical trials of MT with inadequate consideration of blinding tend to systematically magnify the effect of active interventions, thus compromising their overall internal validity and study quality.[6]

To increase the reliability of MT RCTs, proposing an indistinguishable and potentially non-identifiable control intervention that mimics the active intervention without the specific active element and expected therapeutic effect is relevant. Assessing the blinding success of this intervention is also pertinent prior to the start of a trial, since post hoc blinding assessment may be confounded by intervention effects or hunches regarding intervention assignment.[7,8] Given the multiple proposed mechanisms of MT and the well-established biopsychosocial nature of pain,[9,10] the development of credible sensory experiences that mimic active interventions in the field of manual medicine, without therapeutic intent and presumed effects (i.e., inertness), remains an ongoing research priority.

This feasibility trial aims to assess the blinding success of an active MT intervention and a developed sham intervention (i.e., control intervention). This assessment will include i. participant level of certainty regarding the assigned intervention arm, ii. the description of self-reported factors contributing to perceived intervention arm allocation, and iii. level of certainty in outcome assessors regarding participant intervention arm allocation.

2.1 Background and Rationale

Musculoskeletal (MSK) conditions are a rising non-communicable disease and leading cause of years lived with disability (YLD).[11,12] Non-specific low back pain (LBP) represents the most prevalent diagnosis in all age groups, being one of the main reasons for healthcare-seeking and productivity loss in developed countries.[13] Among younger populations, including college-age students, LBP is especially associated with psychosocial factors.[14] In Switzerland, LBP alone accounts for approximately 2.6 billion euros in direct costs, representing 6% of total healthcare expenditures and raising a need for specific treatment.[15]

Although the use of anti-inflammatory drugs is habitual in the treatment of LBP, multiple non-pharmacological interventions are stated as first-line treatment options.[16,17] Among conservative treatment options for LBP, MT remains a non-invasive and cost-effective option, best when used within the context of multimodal care, which tends to include different forms of exercise and psychological interventions. Several plausible mechanisms of MT have been proposed. First, at the level of the peripheral nervous system, mechanical stimuli have been suggested to produce some tissue responses (changes in inflammatory mediators) that can, in turn, produce overall decreased spasms and increased range of motion (ROM).[18] Second, at the level of the spinal cord (central

nervous system), MT has been proposed as a facilitator of certain neuromuscular responses (motoneuron pool, afferent discharges that favor muscle activity), and hypoalgesia (through temporal summation, selective blocking of neurotransmitters). Third, regarding pain-related brain circuits, there are suggested pathways with modulatory effects involving specific brain sections related to stress management such as the amygdala.[18] These modulatory circuits may play a role in subsequent non-specific responses of MT including placebo/expectation, psychological measures (fear, catastrophizing, kinesiophobia), endocrine responses (B-endorphins and endocannabinoid response), and autonomic response (skin temperature, skin conduction, cortisol levels, heart rate).

To elucidate the true efficacy of the active element in MT strategies for back pain and MSK pain in general, the development of validated sham MT interventions with a known degree of blinding success is crucial. This will allow for improved designs of future high-quality controlled randomized controlled trials.

2.2 Justification of Study Intervention

MT, which typically encompasses manipulation, massage and mobilization of joints and soft tissue, may be indicated for acute and chronic LBP.[19,20] MT can be delivered as a stand-alone intervention or in combination with other interventions as an adjunctive yet first or second-line option for LBP management.[21] A standard MT trial of care for a given MSK complaint may involve multiple sessions (i.e., 6–10) for a period of four to six weeks, each of which requires at least 10 minutes to be delivered. There is limited evidence to suggest a definitive optimal frequency or ideal session duration for MT interventions. Pragmatic clinical decisions often take place on an individual basis and consider factors beyond clinical evidence such as patient values and clinician expertise.

2.3 Explanation for Choice of Comparator Intervention

In the context of this trial, the comparator intervention (i.e., control intervention) aims to assess the feasibility and suitability of a sham MT intervention, with respect to blinding success. As exemplified in previous similar studies conducted by Nyugen et al. (2021)[22] and Tavares et al. (2017)[23], the choice of a sham intervention is necessary to control for the placebo effect. The yielded results in blinding success can be used to inform future explanatory trials in the field of MT.

The choice of comparator intervention is further detailed under section *7 STUDY INTERVENTION.*

2.4 Risk / Benefits

Both the defined active and control intervention involve mobilization of defined paraspinal soft tissue regions of the back. Due to the gentle, non-invasive nature of the interventions, potential side effects, if any, are expected to be mild and temporary and may include localized redness, transient muscle soreness, and self-resolving altered local sensitivity.

Potential benefits of MT may include improvement of different dimensions of low back function (i.e., ROM, self-perceived flexibility).[22,23] However, since we will administer a brief MT intervention in our feasibility study, the effect on low back function is uncertain.

2.5 Rationale for Chosen Study Population

The study population is graduate students at the University of Zurich with health-related educational backgrounds. Our study population is limited to 26 students enrolled in the EPI301 course for the Fall 2022 semester. Studies have found that LBP among university students is highly prevalent partly due to prolonged sitting, poor ergonomics, carrying heavy bags, and stress.[24] A study by Anggiat et al. (2018) found a reported LBP incidence of 70% in students, with the majority of surveyed participants reporting prolonged sitting of more than three hours per day.[25] Similar results were observed in a large cross-sectional study of health science students, in which approximately 60% reported lifetime prevalence and 50% reported a 1-year prevalence of LBP.[26] This study additionally found a significant link between sedentary behavior and LBP prevalence.[26]

3 STUDY OBJECTIVES

To assess blinding among participants assigned to either an active or sham-control MT intervention immediately after a one-time intervention session, and to also assess blinding among outcome assesors.

3.1 Primary Objective

The primary objective of this study is to compare an active and control intervention regarding blinding success in participants.

3.2 Secondary Objectives

The secondary objectives are (1) to assess blinding success in outcome assessors of the study, (2) to identify factors contributing to perceived intervention arm allocation in study participants as well as outcome assessors, and (3) to explore the effect of two applied interventions on low back function.

4 STUDY OUTCOMES

4.1 Primary Outcome

The primary outcome of this study is blinding success, as measured by the Bang blinding index (BI).[27] The planned assessment of this outcome is detailed under *0 Assessment of Primary Outcome*.

4.2 Secondary Outcomes

The secondary outcomes of the present study are (1) blinding success in outcome assessors as measured by the Bang BI, and blinding success in both participants and outcome assessors as measured by another validated blinding assessment index—the James BI;[28] (2) different dimensions of low back function; and (3) contributing factors to perceived intervention arm allocation. The planned assessment of these outcomes is detailed under *0 Assessment of Secondary Outcomes.*

4.3 Safety Outcomes

We do not anticipate any risks associated with participation in this study. However, safety outcomes will be evaluated during the trial and participants will have the opportunity to contact the principal investigator to report adverse events at any time. More information on anticipated safety outcomes and their handling during trial can be found under *8.3 Assessment of Safety Outcomes*.

5 STUDY DESIGN AND COURSE OF STUDY

5.1 General Study Design and Justification of the Design

This feasibility trial is a two-arm parallel randomized controlled trial (1:1 allocation ratio) that compares an active and control intervention in terms of blinding success. Based on pre-piloting, enrolled participants will be able to complete outcome assessments and interventions in approximately ten minutes. Individual participants will be assigned a specific time slot during the day of the trial execution. An initial intake form will be filled out by study participants via Research Electronic Data Capture (REDCap 12.5.14)[29] the day before the clinical trial is conducted.

The two tested interventions target the back region and pose minimal risks, even for asymptomatic populations. The active intervention will be operationalized as passive soft tissue mobilization of the lumbar paraspinal muscles with the patient laying in a prone position. The control intervention will be operationalized as a series of bilateral light touches that follow the rhythm of deep breathing of the participant applied at the level of the scapular region and more caudally to the level of the lower part of the rib cage (T12). The control intervention is not known to have any beneficial effects and closely resembles a validated MT sham proposed,[30] thus providing reasonable structural equivalence with respect to the active intervention.

The study population will be restricted to a convenience sample of graduate students from the University of Zurich. There is limited preliminary information regarding the sociodemographic characteristics of this study population and how it may relate to the general graduate student population of Switzerland. It is also unclear how the underlying characteristics of this student population may influence our blinding assessment.

To ensure blinding of participants, outcome assessors and data analysts, the study will be conducted in two different rooms at the University of Zurich. In addition, two different REDCap accounts will be created to prevent accidental unblinding of outcome assessors and data analysts within the REDCap system.

We will perform stratified blocked randomization, with varying permuted blocks (block sizes not disclosed to ensure concealment of allocation), to achieve unconditional exchangeability between the two intervention groups. The stratification variable will be self-reported previous experience with MT coded as a binary variable. A computer-generated randomization sequence created by an EBPI biostatistician not otherwise involved in the trial will be used to randomly assign participants to the active and control intervention groups in a 1:1 ratio. Patients will be allocated to intervention groups through central, web-based randomization (via REDCap) once the demographic data and eligibility criteria are entered in an electronic form and informed consent is obtained. Only trial intervention providers will have information on the assigned intervention.

5.2 Study Duration and Study Schedule

SENSATE will be performed in a single academic center in Zurich (University of Zurich, Main Building) and on a single day (November 8, 2022). For feasible completion, two different rooms will be dedicated to the successful completion of the present study, with further logistic decisions made in accordance with the central organization bodies. The study schedule with the measurements and time points can be found in the Study Procedures Section. Data analysis and course report will be completed by November 14, 2022.

5.3 Methods of Minimizing Bias

To maximize the internal validity of this study and to control for confounding, we will apply multiple methodological approaches. First, we will use stratified randomization to balance the active and control intervention arms regarding the main anticipated confounder (i.e., experience with MT) and to facilitate subgroup analysis.[31] Furthermore, participants, outcome assessors, data analysts and investigators will be blinded to the intervention. Blinding success will be quantitatively assessed. Due to the inherent physical nature of the interventions, the interventionists (i.e., MT providers) cannot be blinded. However, the randomly allocated intervention will not be revealed to them until the time of intervention delivery. The information will be provided on the REDCap platform at the moment the participant enters the intervention room. We will follow standardized instructions from predefined SOPs to ensure that participants in both groups will receive the intended intervention. Furthermore, the SOPs also define the measurement procedure to ensure that the measurement in both groups is applied using the same measures and the same process. Statistical methods to reduce bias from missing data and drop-outs are described in section *10.3 Handling of Missing Data and Drop-Outs.* Finally, we will follow our predefined data analysis plan as described in section 10.

5.3.1 Randomization

Stratified randomization (1:1 allocation ratio) will be performed using previous experience with MT (yes/no) as the stratification variable. This will be done, as we assume this characteristic may play a role when it comes to perceptions about assigned intervention. Varying permuted blocks (block sizes not disclosed to ensure allocation concealment) will be generated by an external biostatistician, and concealment of the list will be achieved electronically in REDCap.

6 STUDY POPULATION

Our study population is made up of health-related graduate students at the University of Zurich. Our study population is limited to 26 students enrolled in the EPI301 Introduction to Epidemiology graduate-level course

for the Fall 2022 semester. Further information regarding our study population can be found in Section *2.6 Study Population*.

6.1 Eligibility Criteria

Inclusion criteria:

- 18 years of age and older
- Students enrolled in EPI301 course at UZH (Fall 2022 Semester)

Exclusion criteria:

- Presence of serious disease of the back (e.g., cancer, severe scoliosis, inflammatory disease, infection)
- Previous spinal operation
- Current pregnancy or breastfeeding
- Inability to lie prone (on the belly)

6.2 Recruitment and Screening

All students enrolled in the "EPI301 Introduction to Epidemiology" course are eligible for participation. Participant eligibility will be determined based on the self-disclosure regarding potential exclusion criteria determined on the day of the study. Study participants will incur no charge upon enrollment into the study nor receive any financial reward for participation.

6.3 Criteria for Withdrawal/ Discontinuation of Participants

Participants will be withdrawn from the study, if one of the following circumstances occurs:

- The participant withdraws from the informed consent.
- The participant wishes to withdraw from the study.
- The participant experiences unexpected adverse events (severe pain or discomfort) during the intervention implementation

7 STUDY INTERVENTION

7.1 General Information

7.1.1 Study Intervention

Participants are randomized to an active or control intervention for low back function. Interventionists will alternate for each participant except in cases where the participant specifically requests an interventionist of a preferred gender.

A) Active intervention

Mobilization of the low back is performed directly by applying MT to the lumbar paraspinal musculature according to a standardized deep soft tissue procedure. Three paraspinal points on each side are stimulated with circumferential movements initiated by the interventionist's hands (reinforced hand placement), lasting 30 seconds each. The total intervention adds up to three minutes.

B) Control intervention

In a standardized deep breathing sequence, patients are instructed to deeply inhale and exhale for 2x3x3 repetitions. Light rhythmic pressure is applied to the thoracic region during expiration with both hands placed on the back, starting at the level of the scapula. The position is adjusted in caudal direction after each repetition. The length of the sham intervention equals the active intervention.

7.2 Compliance with Intervention

Definition of non-compliance:

- Inability to follow study instructions provided independent of the eligibility criteria.
- Interruption or premature termination of the intervention due to significant discomfort

7.3 Data Collection and Follow-up for Withdrawn Participants

Data collection will be performed according to a predefined sequence of questionnaires as well as measurements of ROM (**Table 1**). Withdrawn participants will be excluded from further procedures.

Table 1. SENSATE trial procedures and timing.

Outcome	Measure	Type	T_{-1}	T_0	T_1
Demographics			x		
Experience with professional MT for the treatment of musculoskeletal disability	Have you ever received professional manual therapy for the treatment of musculoskeletal disability?	y/n	x		
	Have you ever provided professional manual therapy for the treatment of musculoskeletal disability?	y/n	x		
Exclusion criterion: back surgery	I have previously had surgery on my back.	y/n		x	
Exclusion criterion: serious conditions of the back	I am currently suffering from a serious condition of the back, e.g., fracture, severe scoliosis, cancer.	y/n		x	
Exclusion criterion: pregnancy or breastfeeding	I am currently pregnant or breastfeeding.	y/n		x	
Self-reported back function assessment	How would you rate your back flexibility at the moment?	5-point Likert scale		x	x
	Do you currently experience ache, pain, or discomfort in your upper back?	y/n		x	x
	If yes: How uncomfortable do you feel with the ache, pain, or discomfort in your upper back?	3-point scale		x	x
	Do you currently experience ache, pain, or discomfort in your lower back?	y/n		x	x
	If yes: How uncomfortable do you feel with the ache, pain, or discomfort in your lower back?	3-point scale		x	x
Thoracolumbar spine flexion–extension	Thoracolumbar angle (Smartphone application)	Degrees of motion		x	x
Blinding success in outcome assessors	To which extent do you know which intervention (active intervention or control intervention) the patient received?	Blinding index			x
Factors contributing to perceptions about assigned intervention (outcome assessors)	Please briefly explain your answer regarding the question above.	Open-ended			x
Blinding success in study participants	To which extent do you know which intervention (active intervention or control intervention) you received?	Blinding index			x
Factors contributing to perceptions about assigned intervention (study participants)	Please briefly explain your answer regarding the question above.	Open-ended			x

Note. MT: manual therapy; y/n: yes–no question; T_{-1}: a day before trial; T_0: pre-intervention; T_1: post-intervention

8 STUDY PROCEDURES

8.1 Study Flow Chart/Table of Study Procedures and Assessments

This study consists of an online survey disseminated on November 7, 2022, (timepoint T_{-1}), followed by an in-person visit taking place on November 8, 2022, during which either the active or control intervention will be administered (**Figure 1**). T_0 specifies the pre-intervention period, T_1 the post-intervention timepoint on the date of the in-person visit. Detailed decision guidelines are summarized in Figure 2.

In an introductory survey, study information will be provided and a number of characteristics will be recorded, such as the name, gender, year of birth, and previous experience with MT. In this same survey, the eligibility of the participants will be verified (**Table 1**).

On the day of the intervention, the intervention protocol, consisting of baseline measurement, application of active or control intervention, and outcome measurement will be performed. The process will take around ten minutes from T_0 to T_1.

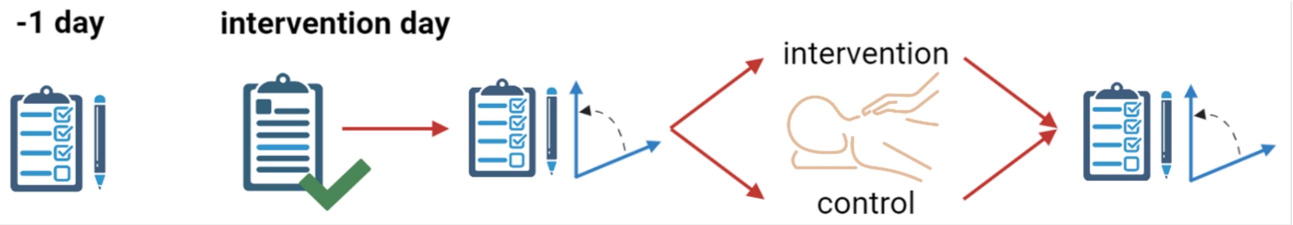

Figure 1. Study procedure of SENSATE: overview of the course of the trial.

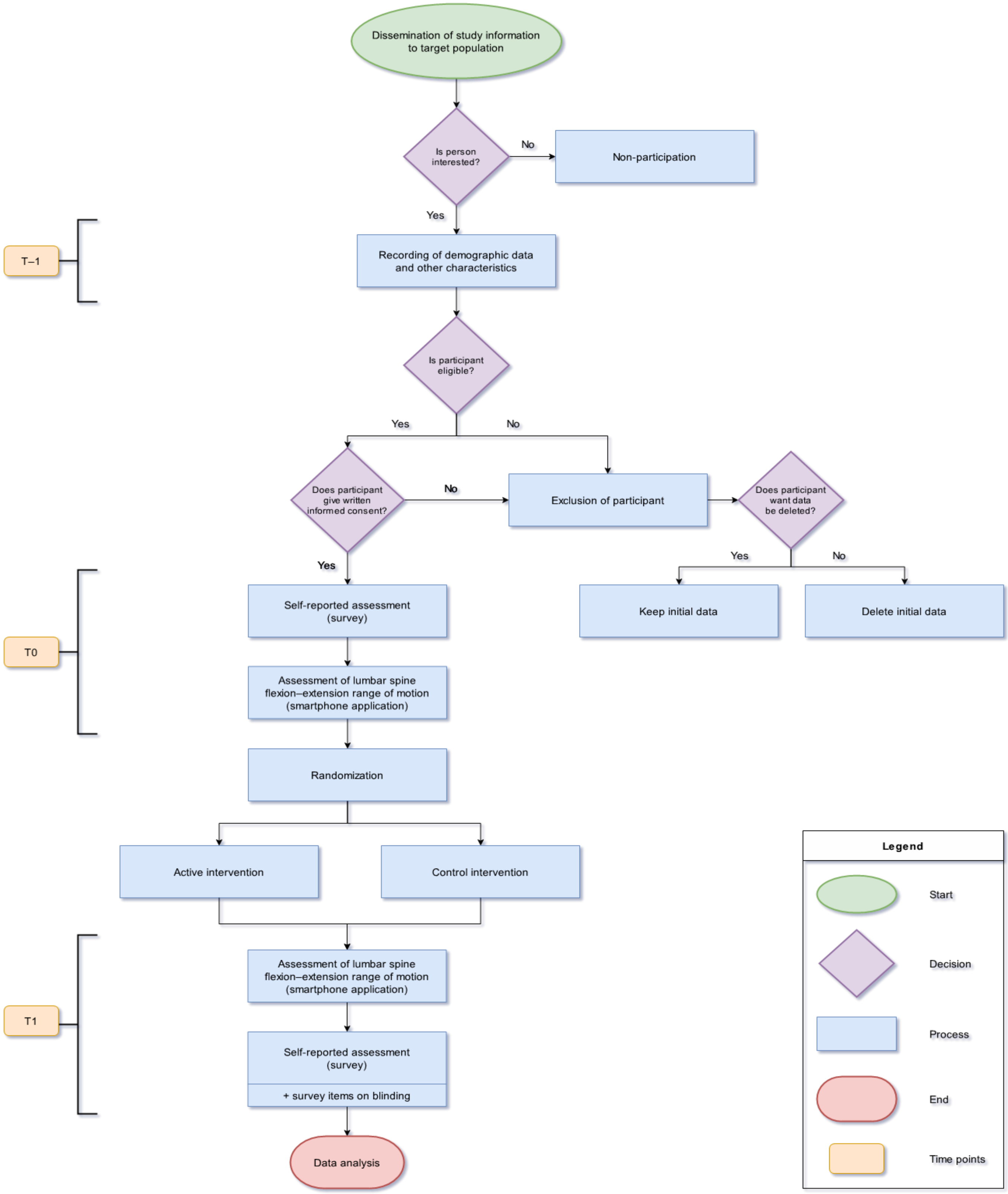

Figure 2. SENSATE study flow.

8.2 Assessments of Outcomes

Assessment of Primary Outcome – Blinding success in study participants

The primary outcome of the present study is blinding success in study participants. This will be measured using the Blinding Index according to Bang.[27] The Blinding Index is derived from a five-point Likert scale inquiring about the degree of certainty regarding perceived intervention arm allocation in study participants (1 = "I strongly believe I received the active intervention"; 2 = "I somewhat believe I received the active intervention"; 3 = "I somewhat believe I received the control intervention"; 4 = "I strongly believe I received the control intervention"; and 5 = "I do not know").

Assessment of Secondary Outcomes

Blinding success in study participants

Blinding success in study participants will be measured with an alternative blinding index – James Blinding Index.[28] Although this index requires different computational considerations, it can be derived from the previously described five-point scale inquiring about the degree of certainty regarding perceived intervention arm allocation (1 = "I strongly believe I received the active intervention"; 2 = "I somewhat believe I received the active intervention"; 3 = "I somewhat believe I received the control intervention"; 4 = "I strongly believe I received the control intervention"; and 5 = "I do not know").

Blinding success in outcome assessors

The Blinding success in the outcome assessors will be measured using the Blinding Index according to Bang.[27] The scale is the same as described for the primary outcome. The question will refer to the degree of confidence of the assessor that participants have received either the active or the control intervention (1 = "I strongly believe they received the active intervention"; 2 = "I somewhat believe they received the active intervention"; 3 = "I somewhat believe they received the control intervention"; 4 = "I strongly believe they received the control intervention"; and 5 = "I do not know").

Factors contributing to perceptions about intervention assigned

Contributing factors to perceived intervention arm allocation will be assessed qualitatively. Participants and outcome assessors will be asked one open-ended question about the reason why they believe that they received/or administered the active or control intervention.

Assessment of the different dimensions of low back function

The low back function dimensions will be assessed using objective and self-reported subjective measures.

ROM assessment: Participant's lumbar spine flexion–extension ROM both pre- and post-intervention will be measured using the pre-installed iOS application called *Measure*® (iOS version 16.0.2, iPhone® model X, Apple Inc., California) following the SOP in Appendix I. Mobile applications were suggested as valid and reliable tools to assess lumbar spine flexion–extension ROM.[32–34] While standing, each participant will be asked to perform maximum spinal extension and flexion with extended knees. No instructions will be provided to participants regarding the velocity of movements. Thus, all participants will perform the movements at their preferred speed, which is known to produce more consistent results of motion characteristics, than pre-defined slow or maximum speed.[35]

Self-reported back flexibility will be measured using an item from the validated International Fitness Scale (IFIS).[36] Furthermore items from the Cornell Musculoskeletal Discomfort Questionnaire (CMDQ) will be used to assess current experience of pain, ache, or discomfort in the upper back as well as in the lower back, and their corresponding intensities.[37]

8.3 Assessment of Safety Outcomes

Serious Adverse Events

Serious adverse events (SAE) will be recorded as part of the outcome measurements. We do not anticipate any risks associated with participation in this study. In case participants experience adverse events, they will be asked to notify the principal study investigator as described in our informed consent form.

Assessments in Participants who Prematurely Stop the Study

Participants not complying with interventions or premature exclusion for other reasons are asked to participate in the outcome measurements at least partly (e.g., only questionnaire). If possible, the withdrawal reason is recorded. There will not be any follow-up visits or assessments.

8.4 Procedures at Each Visit

Intervention visit

Introduction

- Oral information of study protocol
- Signing of informed consent
- Self-assessment of back flexibility of the patient via survey

Baseline measurement

- Measurement of ROM before intervention

Intervention

- Randomized allocation to active or control intervention
- Administration of active or control intervention (see SOP in Appendix I)

Outcome measurement

- Assessment of blinding (questionnaire)
- Measurement of ROM after intervention

9 SAFETY

During the entire duration of the study, all serious adverse events (SAEs) actively reported by the participant in relation to the study intervention are collected and documented in source documents in the case report form (CRF). Study duration encompasses the time from when the participant signs the informed consent until the last protocol-specific procedure has been completed.

9.1 Definitions

Adverse events

Adverse events (AEs) are defined as any untoward medical occurrence in a clinical investigation participant after the intervention. An AE can therefore be any unfavorable and unintended sign (including an abnormal laboratory finding), symptom, or disease temporally associated with the intervention, whether or not related to the intervention. It may also consist of a new disease, an exacerbation of a pre-existing illness or condition, a recurrence of an intermittent illness or condition, a set of related signs or symptoms, or a single sign or symptom.

Serious Adverse Event

A serious adverse event is defined as any event which:

- requires inpatient treatment not envisaged in the protocol or extends a current hospital stay
- results in permanent or significant incapacity or disability

- is life-threatening or results in death or
- causes a congenital anomaly or birth defect.

9.2 Recording and Assessment of Serious Adverse Events

The investigator has the responsibility for SAE identification, documentation, and assessing the causal relationship study intervention. Participants are instructed to actively report any SAE to the study PI.

All reported SAEs will be fully documented in the appropriate *eCRF*. For each SAE, the investigator will provide the onset, duration, treatment required, outcome and action taken with regard to the study intervention.

The assessment by the investigator with regard to the study intervention relation is done according to the following definitions:

Unrelated	• The event started in no temporal relationship to the medical intervention applied and<br>• The event can be definitely explained by underlying diseases or other situations.
Related	• The event started in a plausible temporal relationship to the medical intervention applied and<br>• The event cannot be definitely explained by underlying diseases or other situations.

9.3 Reporting of Serious Adverse Events

If, in the course of a clinical trial, serious adverse events occur in participants in Switzerland, and it cannot be excluded that the events are attributable to the intervention under investigation, the investigator must report these events:

- to the sponsor **within 24 hours** after they become known; and
- to the CEC **within 15 days**.

Safety and protective measures

If immediate safety and protective measures have to be taken during the conduct of this clinical trial, the investigator must notify the CEC of these measures, and of the circumstances necessitating them, **within 7 days**.

10 STATISTICAL METHODS

To assess blinding success within our study, we will use the blinding index (BI) as proposed by Bang et al. and James et al.[27,28] They will be used to assess blinding success of both participants (Bang and James) and outcome assessors (Bang only).

10.1 Sample Size Consideration

A precision-based approach has been used to consider sample size (i.e., width of the 95% CI) for the Bang BI estimates rather than statistical power given this is a methodological feasibility randomised trial.[38] We propose to estimate the Bang BI for each group and present a 95% CI for each mean BI point estimate. For a sample size of 26 participants in total (the maximum given our limited class size), the 95% CI will be the observed BI estimate ± 0.315 points (0.63 points width of the 95% CI) for the primary outcome measure of the group-specific Bang BI, according to Thompson's method (Eq 1) as described by Landsman and colleagues in their study on sample size calculations for blinding assessments.[39] The small class size will inherently limit the precision of our

estimated BI. Ideally, we would want to carry out this trial in a larger study population of approximately 40 participants (20 per group) for a 0.50 points width of the Bang BI 95% CI.

10.2 Planned Analyses

Statistical analysis will be performed with R.[40]

Datasets to be Analyzed, Analysis Populations

The analysis population will consist of all participants included in the study as assigned to the respective intervention, following an intention-to-treat approach. Data analysis will be performed by masking (i.e., pseudonymizing) the intervention arm allocation (active or control) of participant data.

Primary Analysis

Blinding indices BI_i and their respective 95% confidence intervals as defined by Bang et al. will be calculated for each study arm $i = 1$ for active and $i = 2$ for sham

$$BI_i = \frac{\left(2\overline{r}_{i,i} - 1\right) \cdot (n_{i1} + n_{i2})}{(n_{i1} + n_{i2} + n_{i3})}$$

The Bang index ranges from –1 (opposite guessing related to unblinding) to 1 (complete lack of blinding), and can be interpreted as the proportion of participants who guess the intervention correctly over incorrect guesses, including the "don't know" answer category. In the above formula, $\overline{r}_{i,i}$ denotes the proportion of correct guesses across the whole population. Successful blinding in the Blinding Index by Bang et al. was predefined as a value from –0.2 to 0.2 in the bliding index point estimate.[41] This range will also eventually be used in our analysis as evidence of successful blinding.

Secondary Analyses

For our secondary analyses, we will quantitatively evaluate participant's blinding with James's BI. The range of this complementary index ranges from 0 (all correct) to 1 (all incorrect) and does not provide different values according to intervention. An index of 0.5 (half correct, half incorrect) indicates random guessing.[28] In addition, we will also assess blinding index in the outcome assessors using Bang's BI after completing and documenting measurements at T_1.

Both blinding indices (Bang and James) will be calculated with the R package {BI} based on the participant responses about their degree of confidence in their assigned intervention group (displayed in 5-point Likert scale format).

In the secondary analysis, the measured change in ROM (maximum flexion and extension) of the thoracolumbar spine will be compared within and between the intervention groups (active vs. control; T_0 vs. T_1), although formal statistical testing of difference between groups will not be performed, as this would not align with our primary objective. In case a large difference in baseline characteristics is detected between the groups, regression analysis with adjustment of anticipated confounders (i.e., age, gender, experience with MT, care-seeking history for LBP) may be considered.

Contributing factors to perceived intervention arm allocation in study participants, as well as outcome assessors, will be qualitatively analyzed using thematic analysis. Two study investigators will collate collected responses, group them thematically and develop emerging clusters using a consensus-based approach.

10.3 Handling of Missing Data and Drop-Outs

Due to piloted results and data monitoring processes, missing data is unlikely in our study. Data missingness will be further prevented by maintaining active communication channels with study participants before and after enrollment in the study. However, due to our overemphasized small sample size, missing data in quantitative variables will be accounted for using multiple imputation methods, including Multiple Imputation by Chained Equations (MICE).[42] In qualitative variables, the estimated sample size is deemed adequate with respect to anticipated information-richness to perform a thematic analysis.

11 REFERENCES

1. Bang H, Park JJ. Blinding in clinical trials: a practical approach. J Altern Complement Med N Y N. 2013 Apr;19(4):367–9.
2. Dincer F, Linde K. Sham interventions in randomized clinical trials of acupuncture--a review. Complement Ther Med. 2003 Dec;11(4):235–42.
3. Forbes D. Blinding: an essential component in decreasing risk of bias in experimental designs. Evid Based Nurs. 2013 Jul;16(3):70–1.
4. Probst P, Grummich K, Heger P, Zaschke S, Knebel P, Ulrich A, et al. Blinding in randomized controlled trials in general and abdominal surgery: protocol for a systematic review and empirical study. Syst Rev. 2016 Dec;5(1):48.
5. Puhl AA, Reinhart CJ, Doan JB, Vernon H. The quality of placebos used in randomized, controlled trials of lumbar and pelvic joint thrust manipulation—a systematic review. Spine J. 2017 Mar;17(3):445–56.
6. Hohenschurz-Schmidt D, Draper-Rodi J, Vase L, Scott W, McGregor A, Soliman N, et al. Blinding and sham control methods in trials of physical, psychological, and self-management interventions for pain (article II): a meta-analysis relating methods to trial results. Pain. 2022 Jul 11;
7. Bang H, Flaherty SP, Kolahi J, Park J. Blinding assessment in clinical trials: a review of statistical methods and a proposal of blinding assessment protocol. Clin Res Regul Aff. 2010 Jun 1;27(2):42–51.
8. Sackett DL. Turning a blind eye: why we don't test for blindness at the end of our trials. BMJ. 2004 May 8;328(7448):1136.
9. Gevers-Montoro C, Provencher B, Descarreaux M, Ortega de Mues A, Piché M. Neurophysiological mechanisms of chiropractic spinal manipulation for spine pain. Eur J Pain. 2021;25(7):1429–48.
10. Gatchel RJ, Peng YB, Peters ML, Fuchs PN, Turk DC. The biopsychosocial approach to chronic pain: scientific advances and future directions. Psychol Bull. 2007;133:581–624.
11. Vos T, Lim SS, Abbafati C, Abbas KM, Abbasi M, Abbasifard M, et al. Global burden of 369 diseases and injuries in 204 countries and territories, 1990–2019: a systematic analysis for the Global Burden of Disease Study 2019. Lancet. 2020 Oct;396(10258):1204–22.
12. Hartvigsen J, Hancock MJ, Kongsted A, Louw Q, Ferreira ML, Genevay S, et al. What low back pain is and why we need to pay attention. Lancet. 2018 Jun 9;391(10137):2356–67.
13. Buchbinder R, Blyth FM, March LM, Brooks P, Woolf AD, Hoy DG. Placing the global burden of low back pain in context. Best Pract Res Clin Rheumatol. 2013 Oct 1;27(5):575–89.
14. Kennedy C, Kassab O, Gilkey D, Linnel S, Morris D. Psychosocial factors and low back pain among college students. J Am Coll Health. 2008 Sep 1;57(2):191–6.
15. Wieser S, Horisberger B, Schmidhauser S, Eisenring C, Brügger U, Ruckstuhl A, et al. Cost of low back pain in Switzerland in 2005. Eur J Health Econ. 2011 Oct 1;12(5):455–67.
16. Qaseem A, Wilt TJ, McLean RM, Forciea MA. Noninvasive treatments for acute, subacute, and chronic low back pain: a clinical practice guideline from the American College of Physicians. Ann Intern Med. 2017 Apr 4;166(7):514–30.
17. O'Sullivan K, O'Keeffe M, O'Sullivan P. NICE low back pain guidelines: opportunities and obstacles to change practice. Br J Sports Med. 2017 Nov 1;51(22):1632–3.
18. Bialosky JE, Beneciuk JM, Bishop MD, Coronado RA, Penza CW, Simon CB, et al. Unraveling the mechanisms of manual therapy: modeling an approach. J Orthop Sports Phys Ther. 2018 Jan;48(1):8–18.
19. Rubinstein SM, Terwee CB, Assendelft WJJ, de Boer MR, van Tulder MW. Spinal manipulative therapy for acute low-back pain. Cochrane Database Syst Rev. 2012 Sep 12;2012(9):CD008880.
20. Rubinstein SM, Zoete A de, Middelkoop M van, Assendelft WJJ, Boer MR de, Tulder MW van. Benefits and harms of spinal manipulative therapy for the treatment of chronic low back pain: systematic review and meta-analysis of randomised controlled trials. BMJ. 2019 Mar 13;364:l689.
21. Foster NE, Anema JR, Cherkin D, Chou R, Cohen SP, Gross DP, et al. Prevention and treatment of low back pain: evidence, challenges, and promising directions. Lancet. 2018 Jun 9;391(10137):2368–83.
22. Nguyen C, Boutron I, Zegarra-Parodi R, Baron G, Alami S, Sanchez K, et al. Effect of osteopathic manipulative treatment vs sham treatment on activity limitations in patients with nonspecific subacute and chronic low back pain: a randomized clinical trial. JAMA Intern Med. 2021 May 1;181(5):620.
23. Tavares FAG, Chaves TC, Silva ED, Guerreiro GD, Gonçalves JF, Albuquerque AAA de. Immediate effects of joint mobilization compared to sham and control intervention for pain intensity and disability in chronic low back pain patients: randomized controlled clinical trial. Rev Dor. 2017;18(1).
24. Taspinar F, Taspinar B, Cavlak U, Celik E. Determining the pain-affecting factors of university students with nonspecific low back pain. J Phys Ther Sci. 2013 Dec;25(12):1561–4.

25. Anggiat L, Hon WHC, Baait SN. The incidence of low back pain among university students. J -Life. 2018 Oct 2;5(3):677–87.
26. AlShayhan FA, Saadeddin M. Prevalence of low back pain among health sciences students. Eur J Orthop Surg Traumatol. 2018 Feb 1;28(2):165–70.
27. Bang H, Ni L, Davis CE. Assessment of blinding in clinical trials. Control Clin Trials. 2004 Apr;25(2):143–56.
28. James KE, Bloch DA, Lee KK, Kraemer HC, Fuller RK. An index for assessing blindness in a multi-centre clinical trial: disulfiram for alcohol cessation — a VA cooperative study. Stat Med. 1996 Jul 15;15(13):1421–34.
29. Harris PA, Taylor R, Thielke R, Payne J, Gonzalez N, Conde JG. Research electronic data capture (REDCap)—a metadata-driven methodology and workflow process for providing translational research informatics support. J Biomed Inform. 2009 Apr;42(2):377–81.
30. Chaibi A, Šaltytė Benth J, Bjørn Russell M. Validation of placebo in a manual therapy randomized controlled trial. Sci Rep. 2015 Jul 6;5:11774.
31. Kernan W. Stratified randomization for clinical trials. J Clin Epidemiol. 1999 Jan;52(1):19–26.
32. de Brito Macedo L, Borges DT, Melo SA, da Costa KSA, de Oliveira Sousa C, Brasileiro JS. Reliability and concurrent validity of a mobile application to measure thoracolumbar range of motion in low back pain patients. J Back Musculoskelet Rehabil. 2020 Jan 13;33(1):145–51.
33. Pourahmadi MR, Taghipour M, Jannati E, Mohseni-Bandpei MA, Ebrahimi Takamjani I, Rajabzadeh F. Reliability and validity of an iPhone(®) application for the measurement of lumbar spine flexion and extension range of motion. PeerJ. 2016 Aug 23;4:e2355.
34. Kolber MJ, Pizzini M, Robinson A, Yanez D, Hanney WJ. The reliability and concurrent validity of measurements used to quantify lumbar spine mobility: an analysis of an iphone® application and gravity based inclinometry. Int J Sports Phys Ther. 2013 Apr;8(2):129–37.
35. Spindler P, Alzoobi Y, Truckenmüller P, Hahn S, Manzoni YN, Feldmann L, et al. A noninvasive method to quantify the impairment of spinal motion ability in Parkinson's disease. Eur Spine J. 2022 Oct 4;
36. Ortega FB, Ruiz JR, España-Romero V, Vicente-Rodriguez G, Martínez-Gómez D, Manios Y, et al. The International Fitness Scale (IFIS): usefulness of self-reported fitness in youth. Int J Epidemiol. 2011 Jun;40(3):701–11.
37. Hedge A, Morimoto S, Mccrobie D. Effects of keyboard tray geometry on upper body posture and comfort. Ergonomics. 1999 Oct;42(10):1333–49.
38. Bland JM. The tyranny of power: is there a better way to calculate sample size? BMJ. 2009 Oct 6;339:b3985.
39. Landsman V, Fillery M, Vernon H, Bang H. Sample size calculations for blinding assessment. J Biopharm Stat. 2018 Sep 3;28(5):857–69.
40. R Core Team. R: a language and environment for statistical computing [Internet]. Vienna, Austria: R Foundation for Statistical Computing; Available from: https://www.R-project.org/
41. Kolahi J, Bang H, Park J. Towards a proposal for assessment of blinding success in clinical trials: up-to-date review. Community Dent Oral Epidemiol. 2009 Dec;37(6):477–84.
42. Su YS, Gelman A, Hill J, Yajima M. Multiple imputation with diagnostics (mi) in R: opening windows into the black box. J Stat Softw. 2011 Dec 12;45:1–31.
