# Supplementary material for: Is blinding in studies of manual soft tissue mobilisation of the back possible? A feasibility randomised controlled trial with Swiss graduate students
Source: Chiropr Man Therap. 2024 Jan 29;32:3. doi: 10.1186/s12998-023-00524-x (PMC10826218; doi:10.1186/s12998-023-00524-x)
Supplement: Supplementary file 3 — Supplementary Material 3: Study information and consent form [file 12998_2023_524_MOESM3_ESM.pdf]

Study title: Assessing manual interventions of the back in Swiss graduate students: a randomized controlled trial (SENSATE)

Study organised by: Dr. Javier Muñoz Laguna, DC MSc (Investigator)
EPI 301 Introduction to Epidemiology Course
Epidemiology, Biostatistics and Prevention Institute
University of Zurich

Dear candidate participant,

We would like to kindly invite you to participate in the clinical trial **Assessing manual interventions of the back in Swiss graduate students: a randomized controlled trial (SENSATE).** This study examines the effect of two manual therapy interventions on back function. You will find more detailed information about this innovating project in the following subsections.

Aims and general information

The study aims to evaluate the effect of a manual therapy intervention on back function by juxtaposing an active and control intervention. The interventions tested pose very minimal risks and generally take a few minutes to be delivered by trained personnel. As a study participant, you will be randomly assigned (which is the same as flipping a coin) to one of the two different interventions.

Selection

Any graduate student enrolled in the course is eligible to participate in the trial.

Procedure

One day before the administration of the intervention, you will complete a brief survey. The first survey primarily collects demographic information as well as your past experience with manual therapy, and it takes approximately **2 minutes** to complete. Upon arrival to the study location, you will be asked to provide your written and verbal consent to participate in the study. Upon providing consent, a subsequent measurement of the range of motion (flexibility) of your back will occur, and you will fill out a short survey. After this step, you will receive your assigned intervention. **Please note that the study procedures involve physical contact on your back (through clothing) during assessment and intervention. If you feel uncomfortable with physical contact on your back or would prefer an individual of your same gender as the flexibility assessor or intervention provider, you can notify us in advance. All information will be handled confidentially.** After the intervention has been performed, a second flexibility measurement of your back will occur. You will then complete another short survey. Based on pre-pilot testing, the data collection process and intervention will take **around 10 minutes**. To increase efficiency, you will be assigned a time slot as a participant the day before the intervention, and you will be promptly informed via email.

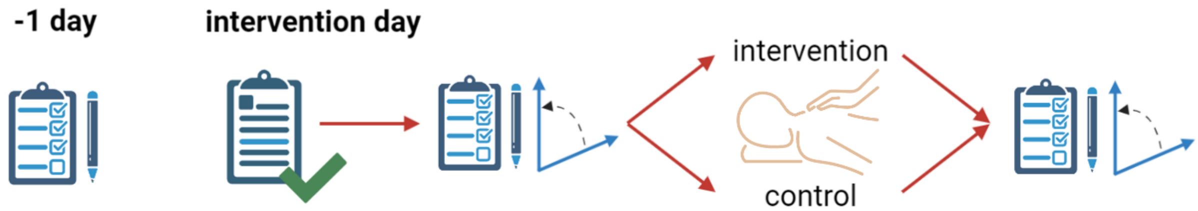

Figure 1. Study procedure: overview of the course of the trial

Benefits

You will not personally benefit from participating in the study.

Obligations

If you consent to participation, we ask you to comply with certain requirements. Successful participation in the study requires the completion of a short survey the day before and the day of the intervention. **We kindly ask that you follow the instructions of the study coordinators. Please note that to maximize our study rigor and collect high-quality data, we ask that you do not share any information on your intervention experience with any other potentially eligible study participant.** To improve the measurement of the range of motion, we also ask you to wear light and tight clothes. For those of you with long hair, we kindly ask you to bring something to tie up your hair.

Risks

We do not anticipate any risks associated with participation in this study. In case you experience adverse events, notify the principal study investigator (JML, listed below).

Data management

Your data, including your full name, will be collected for this study. Upon completing the survey, you will be assigned an automatically generated Study ID. Only the study data manager will have access to it and can connect responses to participants. All data will be stored in a secure location to which only the assigned study staff have access. Database management and survey creation are performed with REDCap. More information on REDCap can be found here. Your data will only be used for the purpose of SENSATE. All persons who have access to your data within the scope of the study are subject to confidentiality. Compliance with data protection regulations will be monitored.

Rights and withdrawal

Your participation in this study is **entirely voluntary** and you can withdraw at any time.

For questions regarding the study, you can reach out to:

Javier Muñoz Laguna, DC MSc
Epidemiology, Biostatistics and Prevention Institute (EBPI, University of Zurich)
Forchstrasse 340, 8008 Zurich
E-Mail: javier.munozlaguna@uzh.ch

Thank you in advance for your participation and cooperation in this stimulating research project.

Sincerely,
The Study Team

With your signature, you consent to participation in this study:

Place, date	Signature of Participant:

Confirmation by the investigator: I hereby confirm that I have explained the nature, significance, and scope of the research project to this participant. I assure that I will fulfil all obligations in connection with this research project in accordance with the law applicable in Switzerland. If, during the research project, I learn about any aspects that could influence the participants' willingness to participate in the research project, I will immediately inform them.

Place, date	Surname and first name of the investigator in block capitals<br>MUÑOZ LAGUNA, JAVIER<br>Signature of Investigator:
