# Supplementary material for: Is blinding in studies of manual soft tissue mobilisation of the back possible? A feasibility randomised controlled trial with Swiss graduate students
Source: Chiropr Man Therap. 2024 Jan 29;32:3. doi: 10.1186/s12998-023-00524-x (PMC10826218; doi:10.1186/s12998-023-00524-x)
Supplement: Supplementary file 4 — Supplementary Material 4: Figure S1 [file 12998_2023_524_MOESM4_ESM.pdf]

Supplementary Material 4

Figure S1. Study interventions and range of motion assessment. The active manual therapy intervention involved a standard soft tissue mobilization protocol (A) applied to six areas of the lumbar paravertebral region (B). The control manual therapy intervention (C) included light touch in six broad areas of the thoracic region and scapulae, distal to the spine (D). Measurement of the active sagittal range of motion started by asking participants to adopt a neutral standing position and placing the inferior limit of the mobile phone (measuring device) at T12, following palpation of the lowermost rib and the T12 spinous process (E). Participants were instructed to reach maximum active flexion and extension, using consistent verbal commands. The degrees of maximum active flexion (F) and extension (G) were then recorded. The measurement sequence ended with the participants adopting a neutral standing position (H).[*]

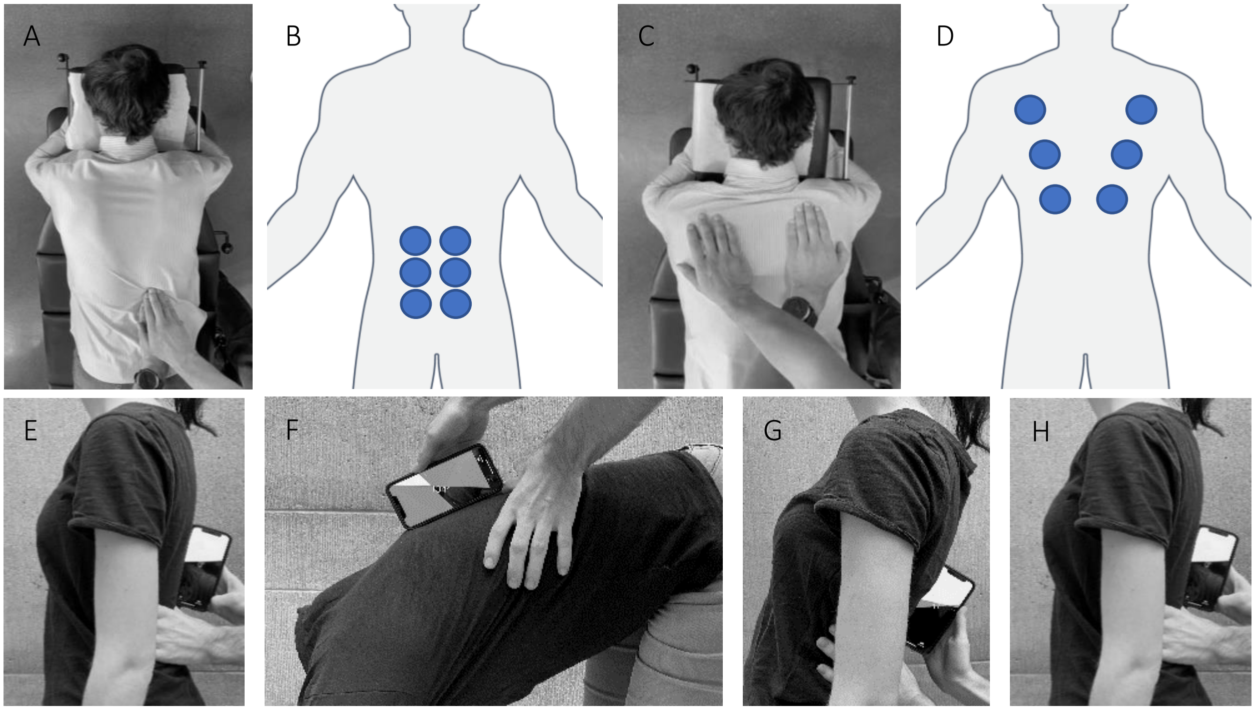

[*] Images for range of motion assessment and interventions depict investigators, whom provided informed consent for publication
