# Supplementary material for: Is blinding in studies of manual soft tissue mobilisation of the back possible? A feasibility randomised controlled trial with Swiss graduate students
Source: Chiropr Man Therap. 2024 Jan 29;32:3. doi: 10.1186/s12998-023-00524-x (PMC10826218; doi:10.1186/s12998-023-00524-x)
Supplement: Supplementary file 5 — Supplementary Material 5: Standard Operating Procedures [file 12998_2023_524_MOESM5_ESM.pdf]

University of Zurich[UZH]

Study title: Assessing manual interventions of the back in Swiss graduate students: a randomized controlled trial (SENSATE)

SENSATE Standard Operating Procedures

Table. Detailed standard operating procedures for the SENSATE trial.

Section	Detailed description of procedures
Entry survey<br>(T_{-1})	- Completion of REDCap survey (collection of demographic data, eligibility criteria, and other characteristics) by study participants<br>- Derive Study ID based on survey completion
Information and informed consent<br>Room 1 (T_0)	- Arrival of participants<br>- Identification of participants using study ID of the survey<br>- Completion of the informed consent form by study participant and assigned study staff in REDCap
Self-reported assessment<br>Room 1 (T_0)	- Completion of baseline survey by study participants<br>- Verbal clarification of gender preferences for interventionists and outcome assessors (if yes: Informing interventionists and outcome assessors via electronic chat)
Range of motion assessment<br>Room 1 (T_0)	- Welcoming participant and presentation of the team (outcome assessors)<br>- If necessary: Ask participant to remove clothes (one layer) and/or tie up long hair<br>- Instructions:<br>➲ Step up to the line<br>➲ Put the feet together<br>➲ Stand in a natural upright position<br>➲ Look straight ahead<br>- Information about the procedure: “I will now take a contact point on your back before I will start to take some measures with the device.”<br>- Taking the contact point for the measurement on the back by palpating the lowest point of the costal arch and placing one thumb on the spine at this level (T12 level)<br>- Positioning the iPhone® with its lower edge on the thumb (contact point)<br>- Calibrating iPhone® (reference level)<br>- Instructions:<br>➲ “Please lean forward as far as possible without bending your knees.”<br>➲ “Please lean backward as far as possible without bending your knees.”<br>- Discharge of study participant<br>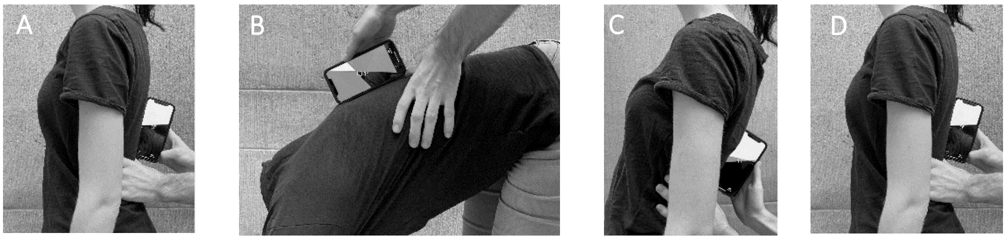<br>**Figure A1**. Position of the iPhone® to measure lumbar spine flexion–extension (A: Start; B: Flexion; C: Extension; D: Stop).

Section	Detailed description of procedures	
Interventions<br>Room 2 ($T_{i\text{-}active}$)	Active Intervention (A)<br>1. Stand in good position on left side of patient<br>2. Place right hand, fingers pointing towards the patient's head, below the lower rib cage on the left paraspinal muscle<br>3. Apply pressure with the left hand and circle on the spot for 30sec<br>4. Release pressure. Remove hands<br>5. Repeat on the left middle lower back and left lower middle back paraspinal muscle part.<br>Repeat on the right paraspinal muscle part on corresponding spots.	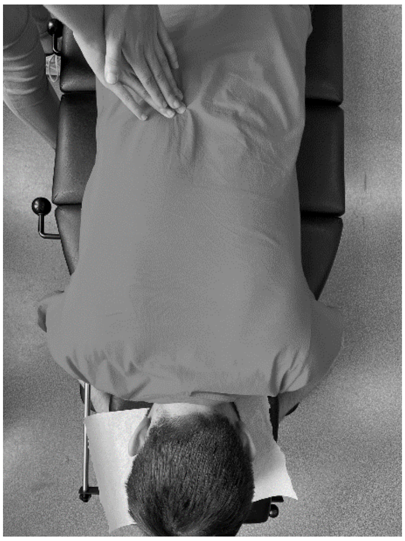<br>Figure A2. Active intervention
Interventions<br>Room 2 ($T_{i\text{-}control}$)	Control/Sham Intervention (B)<br>1. Stand in good position on left side of patient.<br>2. Place hands on scapula with arms crossed (left arm on top).<br>3. Guide breathing and support scapula moving lateral during expiration<br>4. 3x slow breathings<br>5. Move hands caudally for the width of one hand.<br>6. 3x slow breathings<br>7. Move the hand caudally for about the width of one hand. The most caudal breathing sequence will take place at the lower most aspect of the rib cage (~Th12).<br>8. 3x slow breathings<br>9. 30 secs break. No talking. Move to the right side of the patient.<br>Repeat steps 3-9 standing on the right side of the patient.	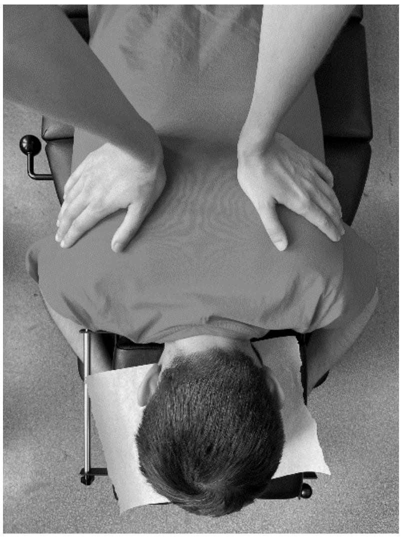<br>Figure A3. Control intervention.
Range of motion assessment<br>Room 1 (T_1)	- Same procedure as T_0	
Outcome assessments<br>Room 1 (T_1)	- Outcome assessors complete independent surveys on their perceptions about study participant’s assigned intervention and factors that contributed to their judgment<br>- Participants complete surveys on self-reported back function measures, perceptions on assigned intervention, and factors that contributed to their judgment	
Trial closure<br>Room 1 (T_1)	- Discharge of study participant	

* Images for range of motion assessments and interventions depict investigators, whom provided informed consent for publication.
