# Supplementary material for: Is blinding in studies of manual soft tissue mobilisation of the back possible? A feasibility randomised controlled trial with Swiss graduate students
Source: Chiropr Man Therap. 2024 Jan 29;32:3. doi: 10.1186/s12998-023-00524-x (PMC10826218; doi:10.1186/s12998-023-00524-x)
Supplement: Supplementary file 6 — Supplementary Material 6: Table S1 [file 12998_2023_524_MOESM6_ESM.pdf]

Supplementary Material 6

Table S1. Bang blinding index estimation table.

Bang blinding index — point estimate[a]	Bang blinding index — variance[a]
The Bang BI point estimate can be estimated by<br><br>$\{2 * (n_{correct} / (n_{correct} + n_{incorrect})\} - 1 *$<br>$\{(n_{correct} + n_{incorrect}/(n_{correct} + n_{incorrect} + n_{don't\ know})\}$,<br><br>where $n_{correct}$, $n_{incorrect}$ and $n_{don't\ know}$ represent the number of correct, incorrect, and 'don't know' responses within an intervention arm, respectively.	The variance for the Bang BI point estimate is given by<br><br>$\{P_{correct} * (1 – P_{correct})+ P_{incorrect} * (1 – P_{incorrect}) +$<br>$2 * P_{correct} * P_{incorrect}\} / (n_{correct} + n_{incorrect} + n_{don't\ know})$,<br><br>where $P_{correct}$ and $P_{incorrect}$ refer to the conditional probability of correct and incorrect responses given a particular intervention assignment, respectively.

[a] Adapted from Bang 2010 [1]

References:

1. Bang H, Flaherty SP, Kolahi J, Park J. Blinding assessment in clinical trials: a review of statistical methods and a proposal of blinding assessment protocol. Clin Res Regul Aff. 2010 Jun 1;27(2):42–51.
