# Supplementary material for: Is blinding in studies of manual soft tissue mobilisation of the back possible? A feasibility randomised controlled trial with Swiss graduate students
Source: Chiropr Man Therap. 2024 Jan 29;32:3. doi: 10.1186/s12998-023-00524-x (PMC10826218; doi:10.1186/s12998-023-00524-x)
Supplement: Supplementary file 7 — Supplementary Material 7: Tables S2, S3, S4, and S5 [file 12998_2023_524_MOESM7_ESM.pdf]

Supplementary Material 7

**Table S2.** Blinding assessment—full results in participants.

5-level response format:

| | Perception | | | | | |
|---|---|---|---|---|---|---|
| Assignment | Strongly believe active | Somewhat believe active | Somewhat believe control | Strongly believe control | Don't know | Total |
| Active MT | 2 (18.2%) | 4 (36.4%) | 0 | 0 | 5 (45.5%) | 11 |
| Control MT | 1 (7.7%) | 3 (23.1%) | 3 (23.1%) | 2 (15.4%) | 4 (30.7%) | 13 |
| Total | 3 | 7 | 3 | 2 | 9 | 24 |

Abbreviation: MT, manual therapy

3-level analysis format:

| | Perception | | | |
|---|---|---|---|---|
| Assignment | Believe active | Believe control | Don't know | Total |
| Active MT | 6 (54.5%) | 0 | 5 (45.5%) | 11 |
| Control MT | 4 (30.7%) | 5 (38.5%) | 4 (30.7%) | 13 |
| Total | 10 | 5 | 9 | 24 |

**Table S3.** Blinding assessment—full results in participants by levels of MT experience.

No MT experience (i.e., participants naïve to manual therapy):

| | Perception | | | | | |
|---|---|---|---|---|---|---|
| Assignment | Strongly believe active | Somewhat believe active | Somewhat believe control | Strongly believe control | Don't know | Total |
| Active MT | 1 (12.5%) | 4 (50.0%) | 0 | 0 | 3 (37.5%) | 8 |
| Control MT | 0 | 2 (22.2%) | 3 (33.3%) | 2 (22.2%) | 2 (22.2%) | 9 |
| Total | 1 | 6 | 3 | 2 | 5 | 17 |

MT experience—providing or receiving:

| | Perception | | | | | |
|---|---|---|---|---|---|---|
| Assignment | Strongly believe active | Somewhat believe active | Somewhat believe control | Strongly believe control | Don't know | Total |
| Active MT | 1 (33.3%) | 0 | 0 | 0 | 2 (66.7%) | 3 |
| Control MT | 1 (25.0%) | 1 (25.0%) | 0 | 0 | 2 (50%) | 4 |
| Total | 2 | 1 | 0 | 0 | 4 | 7 |

**Table S4.** Blinding assessment—full results in outcome assessors.

5-level response formats:

Perception outcome assessor 1 (masked to range of motion measurement)

| Assignment | Strongly believe active | Somewhat believe active | Somewhat believe control | Strongly believe control | Don't know | Total |
|---|---|---|---|---|---|---|
| Active MT | 0 | 0 | 0 | 0 | 11 (100%) | 11 |
| Control MT | 0 | 0 | 0 | 0 | 13 (100%) | 13 |
| Total | 0 | 0 | 0 | 0 | 24 | 24 |

Perception outcome assessor 2 (masked to range of motion measurement)

| Assignment | Strongly believe active | Somewhat believe active | Somewhat believe control | Strongly believe control | Don't know | Total |
|---|---|---|---|---|---|---|
| Active MT | 0 | 4 (36.4%) | 0 | 1 (9.1 %) | 6 (54.5%) | 11 |
| Control MT | 2 (15.4%) | 2 (15.4%) | 1 (7.7 %) | 0 | 8 (61.5%) | 13 |
| Total | 2 | 6 | 1 | 1 | 14 | 24 |

Perception outcome assessor 3 (not masked to range of motion measurement)

| Assignment | Strongly believe active | Somewhat believe active | Somewhat believe control | Strongly believe control | Don't know | Total |
|---|---|---|---|---|---|---|
| Active MT | 2 (18.2%) | 2 (18.2%) | 2 (18.2 %) | 2 (18.2%) | 3 (27.3%) | 11 |
| Control MT | 3 (23.1%) | 2 (15.4%) | 2 (15.4%) | 2 (15.4%) | 4 (30.8%) | 13 |
| Total | 5 | 4 | 4 | 4 | 7 | 24 |

Perception all outcome assessors

| Assignment | Strongly believe active | Somewhat believe active | Somewhat believe control | Strongly believe control | Don't know | Total |
|---|---|---|---|---|---|---|
| Active MT | 2 (6.1%) | 6 (18.2%) | 2 (6.1%) | 3 (9.1%) | 20 (60.6%) | 33 |
| Control MT | 5 (12.8%) | 4 (10.3%) | 3 (7.7%) | 2 (5.1%) | 25 (64.1%) | 39 |
| Total | 7 | 10 | 5 | 5 | 45 | 72 |

**Table S5.** Key subgroup characteristics and outcomes of participants stratified by treatment arm and perceptions about assigned intervention.

| Characteristics and outcomes — immediately post-intervention | Active manual therapy: Correct perception (N = 6) | Active manual therapy: Incorrect perception (N = 5) | Control manual therapy: Correct perception (N = 5) | Control manual therapy: Incorrect perception (N = 8) |
|---|---|---|---|---|
| Age — median (IQR) | 28 (27 to 29) | 29 (27 to 32) | 28 (28 to 28) | 32 (28 to 33) |
| Gender — N (%) | | | | |
| Female | 3 (50 %) | 4 (80 %) | 4 (80 %) | 6 (75 %) |
| Male | 3 (50 %) | 1 (20 %) | 1 (20 %) | 2 (25 %) |
| Other or prefer not to say | 0 | 0 | 0 | 0 |
| Manual therapy experience — N (%) | | | | |
| Providing only | 0 | 0 | 0 | 0 |
| Receiving only | 0 | 2 (40 %) | 0 | 2 (25 %) |
| Providing and receiving | 1 (17 %) | 0 | 0 | 2 (25 %) |
| No experience | 5 (83 %) | 3 (60 %) | 5 (100 %) | 4 (50 %) |
| Upper back ache, pain or discomfort[a] — N (%)* | | | | |
| None | 5 (100%) | 4 (80%) | 4 (80%) | 6 (75%) |
| Yes – slightly uncomfortable | 0 | 1 (20 %) | 1 (20 %) | 2 (25 %) |
| Yes – moderately or very uncomfortable | 0 | 0 | 0 | 0 |
| Lower back ache, pain or discomfort[a] — N (%)* | | | | |
| None | 5 (83%) | 5 (100%) | 4 (80 %) | 8 (100%) |
| Yes – slightly uncomfortable | 1 (17 %) | 0 | 1 (20 %) | 0 |
| Yes – moderately or very uncomfortable | 0 | 0 | 0 | 0 |
| Self-reported back flexibility[b] — N (%)* | | | | |
| Good or very good | 3 (50 %) | 2 (40 %) | 2 (40 %) | 3 (38 %) |
| Average | 3 (50 %) | 2 (40 %) | 3 (60 %) | 5 (62 %) |
| Poor or very poor | 0 | 1 (20 %) | 0 | 0 |
| ROM measurements (deg) — median (IQR)* | | | | |
| ROM, flexion | 125.0 (118.5 to 135.3) | 139.0 (110.0 to 143.0) | 147.0 (144.0 to 148.0) | 136.0 (129.8 to 143.0) |
| ROM, extension | 34.0 (29.8 to 44.3) | 54.0 (46.0 to 56.0) | 40.0 (30.0 to 52.0) | 45.0 (29.0 to 58.3) |
| ROM, total flex-ext | 159.5 (151.0 to 170.3) | 195.0 (153.0 to 197.0) | 178.0 (175.0 to 202.0) | 180.0 (164.8 to 195.0) |
| Change in ROM, flexion | 2.5 (1.3 to 6.8) | 2.0 (-1.0 to 8.0) | 5.0 (3.0 to 9.0) | 2.0 (0 to 4.3) |
| Change in ROM, extension | 0.5 (-1.0 to 4.3) | 1.0 (-2.0 to 6.0) | 0 (0 to 5.0) | 3.0 (-0.3 to 6.3) |
| Change in ROM, total flex-ext | 4.0 (1.5 to 6.5) | 4.0 (-3.0 to 13.0) | 8.0 (5.0 to 9.0) | 7.5 (-0.5 to 9.3) |

Abbreviations: *deg*, degrees; *ext*, extension; *flex*, flexion; *IQR*, interquartile range; *med*, median; *NA*, not applicable; *ROM*, range of motion; *SD*, standard deviation

[a] Adapted from the Cornell Musculoskeletal Discomfort Questionnaire [1]

[b] Adapted from the International Fitness Scale [2]

## References:


1. Kreuzfeld S, Seibt R, Kumar M, Rieger A, Stoll R. German version of the Cornell Musculoskeletal Discomfort Questionnaire (CMDQ): translation and validation. J Occup Med Toxicol. 2016 Mar 25;11(1):13.

2. Ortega FB, Ruiz JR, España-Romero V, Vicente-Rodriguez G, Martínez-Gómez D, Manios Y, et al. The International Fitness Scale (IFIS): usefulness of self-reported fitness in youth. Int J Epidemiol. 2011 Jun;40(3):701–11.
